# Critical Review on the Presence of Phthalates in Food and Evidence of Their Biological Impact

**DOI:** 10.3390/ijerph17165655

**Published:** 2020-08-05

**Authors:** Angela Giuliani, Mariachiara Zuccarini, Angelo Cichelli, Haroon Khan, Marcella Reale

**Affiliations:** 1 “G.d’Annunzio” School of Advanced Studies, “G. d’Annunzio” University of Chieti-Pescara, 66100 Chieti, Italy; info@angelagiuliani.it; 2 Department of Medical, Oral and Biotechnological Sciences, “G. d’Annunzio” University of Chieti-Pescara, 66100 Chieti, Italy; Angelo.cichelli@unich.it; 3 Aging Research Center, Ce.S.I., “G. d’Annunzio” University Foundation, 66100 Chieti, Italy; 4 Department of Pharmacy, Abdul Wali Khan University, Mardan 23200, Pakistan; hkdr2006@gmail.com; 5 Interuniversity Center on Interactions between Electromagnetic Fields and Biosystems, National Research Council-Institute for Electromagnetic Detection of The Environment, (ICEMB-CNR-IREA), 80124 Naples, Italy

**Keywords:** phthalate acid esters, food and beverage contamination, phthalate exposure and health outcomes

## Abstract

Phthalates are a huge class of chemicals with a wide spectrum of industrial uses, from the manufacture of plastics to food contact applications, children’s toys, and medical devices. People and animals can be exposed through different routes (i.e., ingestion, inhalation, dermal, or iatrogenic exposure), as these compounds can be easily released from plastics to water, food, soil, air, making them ubiquitous environmental contaminants. In the last decades, phthalates and their metabolites have proven to be of concern, particularly in products for pregnant women or children. Moreover, many authors reported high concentrations of phthalates in soft drinks, mineral waters, wine, oil, ready-to-eat meals, and other products, as a possible consequence of their accumulation along the food production chain and their accidental release from packaging materials. However, due to their different physical and chemical properties, phthalates do not have the same human and environmental impacts and their association to several human diseases is still under debate. In this review we provide an overview of phthalate toxicity, pointing out the health and legal issues related to their occurrence in several types of food and beverage.

## 1. Introduction

The continuous exposure to different types of chemicals present in the environment and to which humans are exposed during their daily activities may adversely affect human health, and thus represents a global issue. Phthalates (PAEs) are esters of phthalic acid widely spread in many industrial applications, being the main plasticizers used in the polymer industry since the 1930s. They are usually added to plastic materials, such as polyvinyl chloride (PVC), polyethylene terephthalate (PET), polyvinyl acetate (PVA), and polyethylene (PE), at the percentage of 10% up to 60% of PAEs by weight, in order to improve extensibility, elasticity, and workability of the polymers.

PAEs are of great economic and commercial interest thanks to their diverse applications in plastic-based consumer products, such as building materials (flooring and wall coverings, and electric cables), baby toys, clothing, printing inks, packaging materials, pesticides, personal care and cosmetics, pharmaceuticals, as well as medical devices. The structure of the most commonly used PAEs is shown in Table 1.

There is a mounting concern about the ability of PAEs to disrupt hormones and negatively regulate reproductive apparatus [1]. Moreover, due to their environmental persistence and, therefore, bioaccumulation along the food chain, they are considered highly risk pollutants for their negative impact on the environment and living organisms. PAEs can enter food via several routes, i.e., oral, nasal, and transdermal [2]. In addition to their release into the environment during the productive up to the elimination route of plastic-based products, these compounds can easily migrate into food and beverage from various food contact materials during processing, storing, transportation, and preparation. Therefore, in recent years, the diet through PAEs-contaminated food intake and drinking water has been considered the major route of human exposure [3], accounting for more than 67% [4].

In addition to incidental intake of environmental contaminants present in the soil, water, and air, mouthing of phthalate-containing products, other sources of exposure are the dermal route, through skin absorptions from cosmetic and clothing, and intravenous injection [5]. In a recent cross-sectional study, a number of PAEs metabolites have been evaluated in pregnant women’s hair in Crete, namely monoethylhexyl phthalate (MEHP, 68%) and monoisobutyl phthalate (MiBP, 40%) that were likely associated with the use of cosmetics and plastics [6]. Recently, the importance of determining the presence of PAE in food, beverages, and in their packaging has become increasingly evident. Thus, different analytical methods have been developed to determine PAEs in different matrices [7,8,9,10,11,12,13,14,15,16] and many studies have been carried out in order to determine the risk correlated to phthalate contamination in foodstuffs, even though their association with the onset of several diseases is still controversial [17,18,19].

Based on these premises, this review is aimed at provide an overview about the variable presence of phthalates within beverages (alcoholic beverages, soft drink, and mineral water) and food items (edible oils and fats, dairy products, meat and poultry, and edible plants); thus, contributing to the current knowledge and understanding about the migration of phthalates along the food chain, and by taking into account their impact and adverse health effects.

## 2. Physical-Chemical Properties and Applications

Phthalates are a wide class of diesters (dialkyl or alkyl/aryl esters) of ortho-phtalic acid (1,2-benzenedicarboxylic acid) (Table 2) with different physical-chemical properties accounting for many potential uses.

They are manufactured by a reaction of phthalic anydride with various alcohols starting from methanol and ethanol for the smaller compounds, up to iso-decanol straight chain or with some branching [20]. At room temperature, they are almost colorless, odorless oily liquids and are increasingly fat soluble (lipophilic) depending on how long their chain is. Their low melting point and relative high boiling point make them very useful as plasticizer, heat-transfer fluids, and carriers in the polymer industry. Both linear and branched esters are used in the manufacture of plastic, in particular linear esters provide superior flexibility at low temperature and have also lower volatility [21]. According to the length of the R and R’ side chains, they are classified into Low Molecular Weight PAEs (LMW PAEs) and High Molecular Weight PAEs (HMW PAEs).

LMW PAEs include those with 3–6 carbon atoms in their side chain, namely di-n-butyl phthalate (DBP), benzyl butylphthalate (BBP), and di-(2-ethylhexyl) phthalate (DEHP). They are used in PVC products, as well as medical devices, adhesive, paints, printing inks, and enteric-coated tablets. PAEs with shorter alkyl chain, such as di-methyl phthalate (DMP) and diethyl phthalate (DEP), are widely used as solvents and fixatives in fragrances, additives in cosmetics, medical devices, and household and personal care products. DMP and DEP allow perfume fragrance to evaporate more slowly; thus, making the scent linger longer [22].

HMW PAEs with R and R′ from 7 to 13 carbons include mainly di-isononyl phthalate (DINP) and di-isodecyl phthalate (DIDP). They are largely used in industry as plasticizers to increase softness, flexibility, elongation, and durability of rigid polymers such as PVC. They represent the 80% of PAEs used in Europe for plasticized products such as wire and cables, flooring, wall covering, self-adhesive films or labels, synthetic leather, coated fabrics, roofing membranes, and automotive applications. For PAEs with the same molecular weight, branched alkyl chains molecules such as DEHP, BBP, and DINP are very suitable for manufacturing PVC and other resins in order to improve flexibility and general handling properties of the polymer molecules [23]. PAEs act as lubricants because they are bound to PVC through weak electrical bond, reducing the intermolecular forces and viscosity, lowering their glass transition temperature, and thus permitting polymer molecules to slip and slide one another. In PVC materials, the total amount of DEHP, DBP, and BBP used as plasticizers adds up to 30–60% [24,25].

Because of their widespread industrial application, PAEs are ubiquitous contaminants in all the environmental compartments: air (atmospheric aerosols and indoor air), river, marine water/sediments, soil (sludge from sewage and wastewater treatments), and biota [4]. Indeed, they have no chemical linkage with the polymer system and can be lost over time and released into the surrounding environment during production, transport, storage, manufacture, and use and disposal of plastic polymers. The behaviour and fate of PAEs in the environment or in the food chain, as well as their exchange between the different reservoirs depend on a few physico-chemical properties including water solubility (Sw), vapor pressure (Vp), Henry’s-constant (H), air–water partitioning, octanol–air partitioning (Koa), octanol–water partitioning (kow), organic carbon partitioning (koc), their degree of lipophilicity, and abiotic degradation/biodegradation processes [26].

In aquatic system, leaching, drainage, and atmospheric deposition are the major source of PAEs. They are present both in dissolved phase and associated with the Suspended Solid Matter (SSM). DMP, DEP, DBP, BBP, DEHP, and di-n-octyl phthalate (DnOP) are among the most frequently detected in surface water (seawater and freshwater). DBP and DEHP are predominant in fresh and marine water. Biodegradation is the most important process from the removal of PAEs from waters. Thus, the biodegradation of PAEs varies depending on the density and type of species. Generally, they are likely to biomagnify up to the food chain [20]. In sediment, DBP and DEHP are found in abundance. DEP, DBP, and DEHP are predominant in sludge and compost as they bind to organic particles. Microbial action is thought to be the principal mechanism for PAEs degradation both in aquatic and terrestrial systems. PAEs with short alkyl chains are more easily biodegraded and mineralized; however, PAEs with longer alkyl chains could be transformed to compounds with shorter alkyl chains during composting.

In soil, DBP and DEHP are the most abundant PAEs as a result of atmospheric deposition and sewage sludge amendment. According to Vikelsøe et al., there is a correlation between PAEs concentration in soil and the level of sludge amended [27]. Generally, non-cultivated soil contains lowest PAEs, suggesting that these types of pollutants are largely derived from human agricultural activities. Moreover, plastic films in agricultural production, such as plastic sheets and plastic greenhouses, are considered important sources of PAEs in soil.

In air, PAEs are present both in the gas and dust phases. DIBP and DBP are abundant in the gas phase, while DEHP is predominant in the dust phase. More specifically, their concentrations are present at higher levels in urban center as a result of anthropogenic activities [28]. In addition to their impact on the environment, PAEs remain under debate for their toxicity to animals and humans. Some phthalates bioaccumulate and are found in aquatic invertebrates, fish, and amphibians that have lived in phthalate polluted water environments. Numerous studies have focused on the ecotoxicology of PAEs in biota including aquatic organism and rodents, a useful model to investigate toxicity in humans.

The aquatic toxicity of PAEs is strongly influenced by their physical-chemical properties Their Sw, evaluated with the kow, influences their aquatic toxicity, bioaccumulation, and biodegradation. The kow, a measure of lipophilicity, increases as the number of carbon atoms increases on side chain, making PAEs with longer chain more bioaccumulative to organisms. However, high hydrophobic compounds (log Kow > 6) do not follow the same patterns. The acute and chronic toxicity data show that while the lower phthalates (<C6) demonstrate toxicity, the higher phthalates (≥C6) have a reduced toxicity to aquatic organism (fish, algae, and invertebrates) even at concentrations up to the limit of solubility.

To sum up, LMW phthalates display bioaccumulation factors (BAFs) that are greater than predicted from a lipid–water partitioning model and there are specie-specific differences in metabolic transformation capacity across aquatic organisms. On the other hand, PAEs with intermediate molecular weight (i.e., DBP and BBP) have bioaccumulation patterns that are consistent with the general lipid–water partitioning model, whereas HMW phthalates, such as DEHP, tend to have lesser BAFs as a result of tropic dilution in aquatic organisms. At higher log Kow and, consequently, log Koc, chemicals are less absorbed by aquatic organisms; hence, BAFs are reduced resulting from both lower permeability and increased rates of biodegradation or metabolism. Therefore, the ecotoxicity of HMW PAEs is lower than those of LMW PAEs and their effective concentration in body decreases with increasing alkyl chain length [29,30]. PAEs that end up with three to eight carbons in their alkyl side chain have received the most scrutiny since they have been associated to reproductive and developmental effects in lab animals [31].

## 3. Toxicological Aspects and Human Health Effects

**Figure 1:**
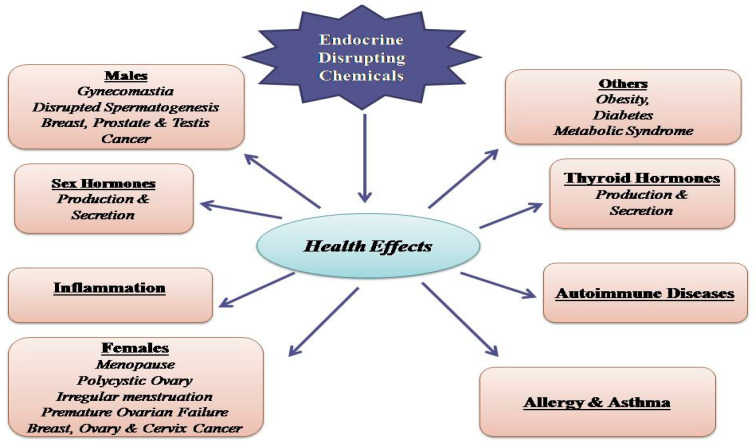
Effects of Phthalates on Human Body.

Widespread exposure to PAEs is posing a great concern regarding their impact on human health. Over the last two decades accumulating evidence suggest that these compounds, upon transformation into primary and secondary metabolites, would act as suspected endocrine disrupting chemicals (EDC), by interacting with different endocrine molecular signaling pathways. Thanks to several methods of human biomonitoring, which allow the detection of biomarkers, see parent compound and their derived metabolites in biological matrices, many researchers tried to highlight the suspected role of PAEs in a wide range of pathophysiological human conditions (Figure 1).

So far, exposure to PAEs has been correlated to a number of health issues, i.e., endocrine and reproductive dysregulation, [32], early puberty, endometriosis, sex anomalies, infertility, altered fetal development, breast and skin cancer, obesity, type II diabetes [33,34], attention-deficit hyperactivity disorder, autism spectrum disorders, cardiotoxicity [35], hepatotoxicity, nephrotoxicity [36], asthma, and allergy [37]. Once absorbed, PAEs undergo chemical transformation via hydrolyzation by esterase or lipase into their respective monoesters or PA and, in a second phase, via sulphonidation or glucuronidation before being excreted [38]. In the attempt to assess the risk referred to phthalate exposure, US EPA and other scientific bodies established a reference dose (the tolerable daily intake; TDI) expressed in microgram (μg)/kilogram (kg) body weight (bw)/day (d) of phthalate as follows: 3500 for mono-methyl phthalate (MMP), 800 for DEP, 100 for DBP, 200 for BBP, 80 for ΣDEHP metabolites, 120 for DINP, and 3500 for DnOP. Phthalate esters (DEHP, BBP, DNBP, and DIBP) are present in the Registration, Evaluation, Authorisation and restriction of CHemicals (REACH) Candidate List within the section “Substances of Very High Concern” (SVHC). That is, levels of exposure to DEHP metabolites (mono(2-ethyl-5-oxohexyl) phthalate (MEOHP), MEHP, and mono(2-ethyl-5-hydroxyhexyl) phthalate (MEHHP)) should be within 20 μg/kg bw/d [39]. It has been estimated that, in normal life, humans are exposed to ≥1.0 g/day of phthalates. As PAEs are quickly metabolized and excreted, the assessment of these compounds in urine is considered appropriate. Noteworthy, children and adults respond in a different manner to PAEs exposure, as a consequence of children’s hand-to-mouth habit that would easily make them ingest DEHP [40,41].

In this work, we reported several epidemiological, as well as in vitro and in vivo studies evaluating phthalate impact in different human health systems, and the putative molecular mechanisms underlying their toxicity.

### 3.1. Phthalates and Endocrine Toxicity

The decline in human fertility over the past decades, putatively associated with environmental causes, has aroused worldwide attention to this issue. Subfertility represents a health and social issue affecting an increased number of individuals, 25–30% of which are males. Interestingly, in a study carried out by Minguez-Alarcon et al., they analyzed, in a population of American males, the correlation between PAEs exposure and the decline of sperm concentration and count of 37% and 42%, respectively, in a specific temporal range (2000–2017) [42]. Many studies reported the role of PAEs in the reproductive toxicity, through the modulation of testicular Leyding and Sertoli cell functions, which are responsible for spermatogenesis, steroidogenesis, and structural/metabolic support of developing germ cells; thus, leading to reproductive failure [43,44,45].

PAEs are considered as endocrine disruptors, being able to negatively modulate hormonal functions and pathways [46,47]; thus, interfering with estrogens and thyroid hormones [48,49]. Indeed, DEHP/MEHP and DBP/BBP/mono-n-butyl phthalate (MBP) can interact with estrogen receptor-1 (ESR1) in humans [50]. Moreover, these metabolites are able to bind to progesterone receptor (PR); thus, competing with endogenous steroid hormones [50].

In males, PAEs can be responsible of the so-called “phthalate syndrome” or “testicular dysgenesis syndrome”, accounting for cryptorchidism hypospadias [44], reduced anogenital distance, altered seminal parameters, infertility [46], and testicular cancer [51]. The molecular mechanism underlying the “phthalate syndrome” might be referred as the ability of these compounds to interact with the hypothalamic-pituitary-gonadal axis (HPG axis) and to take part in signaling pathways involved in steroid homeostasis and biosynthesis [52]. The mentioned syndrome may also occur upon functional impairment of Sertoli, resulting in the inhibition of meiosis, spermiogenesis, and testosterone production by Leydig cells mediated, among others, by oxidative stress [53] and insulin-like growth factor 3(Igf-3) suppression [54]. In particular, it has been shown that cell exposure to MEHP (200 µM for 24 h) triggered an oxidative stress response in rat prepubertal Sertoli cell cultures by increasing lipoperoxides and Glutathione S-Transferases activity while decreasing glutathione levels, and by disrupting adherent cell junction proteins (i.e., N-Cadherin, occluding, ZO-1, and catenin) [53]. Accordingly, it is plausible that DEHP, at doses of 500 mg/kg or more, causes atrophy of seminiferous tubules and decreased ATP-dependent sperm motility, inhibiting DNA replication, decreasing SIRT1, and inducing ROS-mediated apoptotic cell death [55]. ROS overproduction is considered a major player in sperm dysfunction, as assessed by the significant increase in malondialdehyde (MDA) formation (derived from lipid peroxidation and a marker of oxidative stress) in the testis following DBP treatment in male rats [56]. Noteworthy, ROS are thought to disrupt plasma membranes of the sperms that are rich of highly sensitive polyunsaturated fatty acid [57], to decrease testosterone levels and to elicit apoptosis of spermatogenic cells and disruption of their mitochondrial membranes; thus, impairing sperm quality [58]. Furthermore, they observed a DEHP-elicited phenotypic testicular alteration in vivo [59].

Another PAE metabolite, namely DBP, has been demonstrated to induce testicular toxicity in rats [56]. Indeed, oral treatment with increasing doses of DBP (0, 200, 400, or 600 mg/kg/day for 15 consecutive days) in male rats caused a decrease in sperm count in the epididymis, amount of sperm in the testes, likely due to a decrease in serum levels of follicle-stimulating hormone as well as levels of testosterone and activity of testicular lactate dehydrogenase activity, the latter being a crucial enzyme for Sertoli cells to produce ATP necessary for spermatozoa motility and to prevent apoptosis of testicular germ cells. A dysfunction of testicular activity is likely due to PAEs-mediated decrease in levels of serum testosterone, as well as other key regulators of sperm production, namely, follicle-stimulating hormone (FSH) and lactate dehydrogenase (LH). A recent work reported a positive correlation between MEHP and FSH/LH [60].

In a recent study, the role of PAEs as modulator of sperm epigenetic modification has been investigated [61,62]. Specifically, low-doses of PAEs (MMP, mono-ethyl phthalate (MEP), MBP, monobenzyl phthalate (MBzP), MEHP, and MEOHP in the range from 0.85–20.53 µg/g), measured in urinary samples of selected male participants, were analyzed by multiple linear regression models to assess the impact of these compounds on semen quality parameters. Interestingly, they found that while several PAEs correlated with sperm motility, the latter was negatively regulated by DNA hypermethylation. It is well known that DNA hypomethylation plays a crucial role in spermatogenesis by modulating the expression of developmental genes and it is positively associated with higher quality of sperm [63]. On the contrary, DNA hypermethylation may cause oligoasthenoteratozoospermia due to abnormal chromatin/DNA integrity [64]. Another epigenetic mechanism by which PAEs would exert their endocrine disruption includes abnormal hypomethylation of paternally imprinted H19 gene and hypermethylation of maternally imprinted LIT1 gene [65]. A plausible explanation may rely in the PAEs-mediated oxidative stress that would prevent the interaction of methyl CpG-binding proteins to the CpGs; thus. leading to DNA demethylation [66].

It has been reported that direct or indirect maternal exposure to DEHP decreases in utero expression of mineralocorticoid receptor (MR) in rat Leydig cells [67] as well as in the expression of fetal testicular mRNA levels of 17α-hydroxylase and cytochrome P450 17A1, all accounting for reduced testosterone levels in adult rats [68]. However, in a study carried out in Germany by Herr et al., the increased exposure to DEHP metabolites (40.56 μg/L) was not correlated to altered semen profile [69].

In women, it is possible to detect phthalates from different biological matrices [70,71]. Furthermore, unconjugated PAEs, namely DEHP, DEP, DBP, BBP, MEHP, MEHHP, MEP, MBP, and MBzP, are also able to cross the placental barrier; thus, affecting post/pre-natal development [72]. Of note, exposure to phthalates, mainly monoesters, correlates with reduced gestational age of fetus [73], follicular atresia [74], endometriosis [75], infertility [76], and pubertal development [77] increased birth loss [19], reduced yield of oocytes [78]. Maternal exposure to DEHP (0, 50, or 200 mg/kg) during pregnancy caused a fetal growth restriction and lowered placental weight in a gender-independent manner [79]. The inhibition of placental cells’ proliferation likely involves the MEHP-mediated decrease of progesterone receptor level, which in turn would cause the down-regulation of Cyclin D1 and induce progesterone synthesis [80].

These compounds have been demonstrated to cross the human placenta and reach the umbilical cord [81] and the amniotic fluid [82]. The altered placental development might be due to a peroxisome proliferator-activated receptor (PPAR)γ-mediated disruption in placental lipid metabolism, accounting for modified glycerolipids and glycerophospholipids levels, with a marked accumulation of triacylglycerols [83]. Furthermore, the presence of DEHP was also detected in maternal milk; thus, exposing newborns to these contaminants during breast feeding [84]. In mice, DEHP administration between the weaning period and maturity has been shown to disrupt ovarian function and decrease the expression of follicular development factors (i.e., C-KIT, KITL, GDF9, and ATM), as well as ovarian microRNAs (miR-17-5p, let-7b, miR-181a, and miR-151) that are responsible for inhibition of follicular granulosa cell proliferation and for bax/bcl2-mediated apoptosis [32].

The effect of phthalates on women reproductive system likely relies on MEHP formation [85]. Importantly, PAEs can mimic hormone activity through the binding to a number of human receptors. They bind to hERα,β, thus eliciting either estrogenic or anti-estrogenic effects [86]. DEHP would be able to decrease expression of the Arom gene and, consequently, E2 levels in vitro; thus, affecting follicle growth [87]. Moreover, they form a complex with human peroxisome proliferator-activated receptor α, β or γ subtypes (PPARs) and, in turn, interact with follicle stimulating hormones. This indirect effect would provoke estradiol inhibition and suppression of aromatase as a consequence of cyclic adenosine monophosphate (cAMP) decrease in granulosa cells [88]. Finally, they are able to regulate aryl hydro-carbon receptor (AhR) as well as the activity of metabolic enzymes involved in ER metabolism [89]. PAE have been also taken into consideration as risk factors for thyroid endocrine system disruption [90]. Furthermore, as thyroid system is strongly connected to the reproductive one, an association between urinary concentrations of PAEs and thyroid hormones has been investigated in a cross-sectional study [44]. The study outlined the inverse relationship between higher doses of MEP or MEHP and lower serum free thyroxine (FT4) or serum thyroid-stimulating hormone (TSH), respectively.

In juvenile rats were sub chronically exposed to low doses of DEHP (0.3–3 mg/kg) from their weaning to maturity, this compound significantly increased expression of genes related to thyroid regulation, namely thyrotropin releasing hormone (Trh) parathyroid hormone (Pth) in females and thyroid hormone responsive (Thrsp) in males. On the other hand, higher doses of DEHP (30 and 150 mg/kg) were shown to induce hyperplasia and hypertrophy of thyroid glands [91].

According to the human biomonitoring study from the national health and nutrition examination survey (NHANES, 2007–2008), urinary DEHP is negatively associated with total thyroxine (T4), free T4, and total triiodothyronine (T3), whereas positively associated with thyroid-stimulating hormone (TSH) [92]. Conversely, among the adolescents, DEHP metabolites correlated with T3 concentration. In contrast with these findings, Baralić et al. and Sun et al. have not found a significant relationship between oral exposure to DEHP (50 mg/kg bw) in rats for 28 days and T3 or T4 serum levels, although the same parameters decreased at higher doses of DEHP (500 mg/kg b.w.) [93,94]. The reduction of serum thyroid hormones is likely due to a DEHP-mediated modulation of biosynthesis, biotransformation, biotransport, TSH receptor levels, and metabolism of thyroid hormones.

### 3.2. Phthalates and Cancer

Phthalates have been extensively correlated to several human cancer, i.e., skin, liver, prostate, and breast cancer [95,96,97]. Noteworthy, PAEs (i.e., BBP and DEHP) would increase the expression of vascular endothelium growth factor (VEGF) and, consequently, angiogenesis and tumor progression in breast cancer cells [98]. In a Mexican study (N = 233), they detected significantly higher MEP concentration (169.58 g/g creatinine) in women with breast cancer compared to healthy ones (106.78 g/g). One possible mechanism might rely on the ability of PAEs to provoke DNA damage in mammary epithelial cells [99], and on the induction of PPARs signaling associated to BARC gene activation [100]. In both hepatic and breast carcinoma, PAEs likely induce Phosphoinositide 3-kinase (PI3K)/Protein kinase B (PKB) or cyclic adenosine monophosphate (cAMP)-Protein Kinase A (PKA)- cAMP response element-binding protein (CREB) signaling cascades, the latter responsible for the activation of the AhR-evoked proliferation of mammary cancer cells [101,102]. In particular, the increased Histone Deacetylase 6 (HDAC6) expression induces the activation of the nuclear β-catenin-lymphoid enhancer binding factor 1 (LEF1)/T-cell factor-4 (TCF4) transcriptional complex and, in turn, that of the oncogene c-Myc [103]. Interestingly, in a study carried out by Ito Y. et al., they observed a PPARα-independent effect of phthalates that would elicit hepatic cancer via c-jun/c-fos/Activator protein-1 (AP1) signaling [104]. In addition, it has been hypothesized that phthalates deregulate several miRNAs involved in breast cancer progression (i.e., miR-34b-5p, miR-7686–5p, and miR-1291) [105].

As one of the PAEs’ target is thyroid, these compounds seem to play a major role in the onset of thyroid cancer, being able to activate estrogen receptor, induce VEGF-mediated angiogenesis [106].

Noteworthy, several PAEs (i.e., DEHP, BBP, and DBP) are suspected to interfere with cell cycle-related genes responsible for prostate cancer cell proliferation [97]. Indeed, PAEs would promote the growth of PC3 and 22RV1 prostate cancer cells via up-regulation of MAPK, c-fos, and c-Jun, three proteins involved in AP1-mediated cell proliferation. Collectively, numerous data from literature revealed that exposure to phthalates would exert a tumorigenic activity through the activation of different signaling pathways (cAMP/PKA/CREB, PI3K/PKB, c-jun, HDAC6/c-myc) mediated by their interaction with AhR, PPAR, and ER, beyond a putative epigenetic modulation.

### 3.3. Phthalates and Metabolic Disorders

It is well known that insulin resistance is a common feature of many diseases, i.e., type-2 diabetes (T2D), atherosclerosis, and non-alcoholic fatty liver disease (NAFLD) [107,108].

Among other compounds, DEHP and its metabolites have been correlated to the onset and progression of (T2D) [33]. In a study carried out in USA, MBP has been associated with poor insulin secretion, and MEP and MMP to insulin resistance assessed by Homeostatic model assessment of insulin resistance (HOMA-IR) index [109,110]. In a cross-sectional study (Canadian Health Measures Survey (CHMS, 2009–2011)) carried out by Dales et al., the authors measured the association between PAEs exposure (from urinary PAEs metabolites) and a number of parameters such as fasting blood glucose, glycosylated hemoglobin (HbA1C) levels, and insulin [111]. They found a possible link between PAEs exposure and increased concentration of pre-diabetes-related markers. Indeed, MBzP, mono-(3-carboxypropyl) phthalate (MCPP), MEHHP, MEHP, MiBP, and total DEHP metabolites correlated with increased HbA1C and reduced blood glucose control. Moreover, DEHP metabolites were correlated to increasing fasting glucose, insulin, increase of HOMA-IR of 0.15 (95% CI 0.04, 0.26) and of HOMA-β of 10.24 (95% CI 3.71, 16.77). In a recent study, they investigated the putative role of PAEs in insulin resistance and risk for the development of obesity and NAFLD, the latter being the hepatic manifestation of metabolic syndrome [112]. The results showed that PAEs correlated with hyperglycemia—a risk factor for early phase NAFLD according to [113]—in all the examined groups of both genders, namely patients affected by obesity or type-2 diabetes mellitus, and even non-obese non-diabetic volunteers. The derived parameters, namely triglycerides glucose (TyG) index [114] and TyG-BMI, considered as prognostic markers of insulin resistance and NAFLD in non-obese individuals [115], were found positively related to MEP exposure in non-obese healthy volunteers [112].

Noteworthy, the modulation of primary and surrogate markers in all the above studies, from anthropometric parameters to glycemia or insulin resistance (HOMA-IR), reflect the need to evaluate them not as individual markers but, in a wider perspective as potential risk factors for early identification of type-2 diabetes mellitus, atherosclerosis, and cardiovascular diseases [116]. The hypothesis of phthalate-induced insulin resistance relies on their ability to induce mitochondrial dysfunction and oxidative stress, leading to the onset of the disease [117]. Several epidemiological studies also reported an obesogenic activity of PAEs (i.e., MEP, MEHP, MBzP, MEHHP, and MEOHP), which seems to depend on age and gender [118]. According to Buser et al., low MW phthalate metabolites (MBP, MEP, and MiBP) were able to cause obesity in male children and adolescents whereas high MW phthalate metabolites (mono(2-ethyl-5-carboxypentyl) phthalate (MECPP), MEHHP, MEOHP, MEHP, MBzP, monocarboxy-isononly phthalate (MCNP), and monocarboxyoctyl phthalate (MCOP)) and DEHP (MEHHP, MEOHP, MEHP, and MECPP) contributed to obesity in all adults [119]. In a Chinese study, children exposure to MEHP elicited an increase in the body-mass index (BMI) and waist circumference. Interestingly, MEHHP and MEOHP resulted to be correlated to BMI in the 8–11-year age group [120].

The national Puberty Timing and Health Effects in Chinese Children (PTHEC) study reported a correlation between environmental PAEs exposure and metabolic changes (i.e., obesity and overweight) in children (OR = 1.586, 95% CI: 1.043, 2.412) [121]. The presence of different PAE monoesters (MMP, MEP, MBP, MEHP, MEOHP, and MEHHP), measured with an electrospray triple quadrupole mass spectrometer (ESIMS/MS) and revealing the metabolomic profile of urine samples, corresponded to higher concentration of metabolic markers related to disrupted arginine and proline metabolism and fatty acid reesterification (monostearin, 1-monopalmitin, stearic acid, glycerol 3-phosphate, 5-methoxytryptamine, d-alanyl-d-alanine,pyrrole-2-carboxylic acid, and butyraldehyde); thus, contributing to the onset of overweight and obesity in school-age children. Of note, a link between visceral obesity (measured as waist circumference and waist-to-height ratio) and MEHP was also found in healthy normal-weight individuals [122,123].

Another study comparing PAEs and metabolic syndrome was a cross-sectional study from the National Health and Nutrition Examination Survey (2003–2014) data carried out by Gaston and Tulve [124]. The authors observed the putative association between the presence of main urinary PAEs’ metabolites (i.e., MiBP, MEP, MBP, MBzP, DEHP, and MCPP) and risk factors for the metabolic syndrome; data were also adjusted for the socioeconomic status of adolescents (mean age = 16 y.o.), although this variable did not affect the overall findings. The results revealed that the prevalence of metabolic syndrome in the total population of adolescents was 5.3%, being males but not females with higher MnBP concentrations affected to a greater extent by dyslipidemia. The latter has been also associated with MEHP exposure in other studies enrolling both obese and healthy adults [112], and consisted in increased triglyceride and decreased low high-density lipoprotein (HDL) cholesterol serum levels, likely due to the lipolysis in the adipose tissue, followed by the entering of free fatty acid to the liver and the hepatic efflux of triglycerides and hyperlipidemia [125].

As PPAR are known to be key players in lipid and glucose homeostasis [126], it is plausible that phthalate involvement in metabolic disorders likely correlate to their binding to PPAR-α, γ, the latter associated to adipogenesis, and controlled by neuroendocrine pathways involved in the hypothalamic-pituitary-adrenal axis [127], but also to other receptors such as steroid hormone receptors, thyroid hormone receptors, retinoid X receptors, liver X receptors, and farnesoid X receptors [107]. Specifically, MEHP can interact with PPAR-α and -γ and induces PPARγ adipocyte differentiation as well as the selective activation of different PPARγ co-regulators including Mediator 1 (Med1) and Peroxisome proliferator-activated receptor gamma coactivator 1-alpha (PGC-1α), but not p300 and Proto-oncogene tyrosine-protein kinase Src (SRC) [128]. Other mechanisms linked to phthalate-induced adipogenesis may involve their interaction with thyroid hormone channels, androgen and estrogen receptors, and pregnane X receptors, all linked to lipogenesis [123,129].

### 3.4. Phthalates and Neurotoxicity

Although very few studies have been carried out on their neurotoxic effects, phthalates putatively affect the onset of several neurological disorders [130].

They observed that early-life PAEs exposure (i.e., DEHP, MEP, and MCPP) was able to negatively affect cognition (child IQ) especially at age 3 years, whereas no proof of association was detected during gestation or in >3 years old children [131]. Another study provided evidence of a DINP-mediated negative effect in child psychomotor skills following prenatal exposure [132].

Of note, DEHP would induce teratogenic effects (disruption of normal fetal brain development) due to its ability to cross the placenta [133,134]. Low-dose DEHP exposure (50 and 200 mg/kg/d) decreased the levels of the N-methyl-d- aspartic acid (NMDA) receptor subunits NR1 and NR2B in the hippocampus in offspring mice; thus, contributing to impair spatial learning and memory [135]. MnBP (46.7 g/L urine), but not MEHP (3.4 g/L), have been also correlated to attention-deficit hyperactivity disorder (ADHD) in humans [136].

Accordingly, exposure to PAEs seems to provoke behaviors overlapping with ADHD, namely emotional hyperreactivity, aggression, and impairment in working memory [137,138].

In the U.S. children (N = 1493) of 6–15 years of age, with reported attention deficit disorder (ADD) or Learning Disability (LD), it has been demonstrated an association between phthalates and these disorders, with a prevalence in girls than boys [139]. The mechanism underlying this effect may rely on the disruption of thyroid system during pregnancy [140,141].

Neurotoxicity may also derive from phthalates-induced ROS production and down-regulation, in hippocampus, of brain-derived neurotrophic factor (BDNF), a key player in dendrite outgrowth and synaptic plasticity associated to cognitive and learning functions. They observed that low-dose DEHP exposure (10 mg/kg) decreased dorsal hippocampal BDNF expression and dendritic spine density [142].

### 3.5. Phthalates and Immune System

Phthalates comprise a group of xenobiotics that have been shown great effects on immunological system [143], by mimicking natural hormones that are responsible for the normal functioning of the body like development, reproduction, homeostasis, and behavior.

When the impact of phthalates on immune responses was evaluated no consistent results emerged, in fact, several studies have reported the potentiation of immune responses or inflammatory reactions, other studies were unable to show effect, while other studies have shown inhibitory or immunosuppressive effect [144,145,146]. Few studies have estimated the impact of phthalates on the Th1/Th2 balance and their cytokine products. Increased production of IL-4, by Th2 cells, was described by Lee and Maruyama [145,147]. Badr et al. reported that dietary exposure of rats to DEHP shift the Th1/Th2 cytokines balance towards Th2 type phenotype, with a liver protection against Th1-response induced by Mycobacterium bovis protein [148]. The study by Greene et al. reported that Di-(2-ethylhexyl)phthalate (DEHP) may act directly on Mɸs increasing chemokine and cytokine gene expression altering their responses, and in women may drive alteration of uterine and/or MΦ factors involved in develop of endometriosis [149]. Studies that have investigated the influence of phthalates on cytokine secretion by primary human peripheral blood mononuclear cells (MNC) and lymphocytes T showed that phthalate diesters influence cellular signal pathways that lead cytokine production, enhancing the secretion of interleukin (IL)-6, IL-10 and the chemokine CXCL8 and impairing release of tumor necrosis factor (TNF)-α, IL-2, IL-4, and interferon-γ [150], which could hypothetically prime a decreased synthesis of antibodies, albeit impact of phthalates on cytokine expression was nor confirmed in other studies [143,151]. Other immune parameters, such as weight of lymphoid organs, thymus histology, and antibody levels were evaluated and no effects [151] or immunosuppression were observed [152]. The oral administration of DnOP at low doses to albino mice caused significant pathomorphological and immunological alteration [153]. Studies in experimental animals that focused on effects of phthalates on inflammatory processes suggest enhanced inflammatory responses and increased chemokines expression [154]. Phthalates inactivate peroxisome proliferator-activated receptor-γ (PPAR-γ), a nuclear transcription factor that mediates the resolution of inflammation, MEHP was able to inhibit chemotaxis, induce oxidative metabolism, stimulate the production of IL-1β and VEGF, and inhibit production of MIP-1β [155,156].

In addition, the in vitro effects of phthalates were evaluated using both cell lines and primary immune cells, yielding conflicting results on expression of cytokines [145] or decreased macrophages production of nitric oxide and tumor necrosis factor, pointing out an immuno-suppressive effects [157,158], or apoptosis in B cells, suggestive of down-regulation of antibody responses [159,160].

Many studies have highlighted that adjuvant-like properties of phthalates may be responsible for increased risk of development of allergies and asthma [37], and MEHP, MNOP, and MINP, at different doses, showed immunosuppressive and adjuvant effects [161]. Jepsen et al. showed the structure-related intensity of adjuvancy; thus, monophthalates may be weak or potent cytokine inducers, and that several monophthalates could increase of IL-6 and IL-8 concentration-dependently, while at high concentrations all phthalate suppressed cytokine production [162].

Previous studies have shown that phthalates with eight carbon atoms in two esters group, total having sixteen carbon atoms that may be unequally distributed in the esters group, did not affect its adjuvant activity, and thus may have the highest adjuvant effect. Phthalate metabolites and plasticizers boosted the effect of immunogens [161]. The structure–activity relationship (SAR) studies of immuno-stimulatory effects of the most commonly used phthalate plasticizer DEHP revealed that the minor alteration in Phthalates structure results in remarkable affects in adjuvant activity. Such types of effects are defined as the inborn ability of compounds to enhance the humoral immune response [163]. Thus, the serum concentration of antibodies was investigated in several studies [143,145,146]. Furthermore, the physiochemical and stereo chemical properties highly affected the adjuvant activity of Phthalates and it is limited to formation of IgG1 antibody. In addition to this, it is also noted that phthalates also play a key role in elicitation phase of allergy [164]. The study by Larsen and Nielsen revealed that no effect was observed on IgE antibodies [144] or IgE allergy promoting effects of DEHP [165], as well as its key metabolites, MEHP [166]. It is well know that chronic low-grade inflammation contribute, together with immunosenescence, to neurodegenerative diseases [167], and that phthalate modulating molecular signaling pathways that underlie inflammation and inflammation-related disease risk, may play a key role in the promotion of inflammatory activity via multiple mechanisms.

## 4. Biochemical Regulation of Phthalates Effects

As Phthalates belong to group of EDCs that have the capability of changing immune response through various mechanisms. Of them the most commonly studied receptors are estrogen receptor (ER), estrogen related receptor (ERRs), Peroxisome Proliferator-activated Receptor G (PPAR-G), TLRs, and NLRs. Studies revealed that phthalates change the level of cytokines by mediating through estrogen receptors [168]. An in-vitro study revealed that DINP altered the activation of mitogen activating protein kinase (MAPK) signaling pathway through an estrogen receptor (ER) dependent pathway [169]. The in vivo studies showed that in males rats the ERa gene expressions were reduced as compared to females’ first and second generations offspring, which is associated with low level of IL-2, IL-12, IFN-g, and TNF-a in spleen [170]. In continuation with these proceedings, a new study was reported that first and second male generations offspring have low ERa gene expressions in islets which is linked with augmented proinflammatory cytokine levels in pancreatic lysates [171]. Thus, it is considered that ERa has anti-inflammatory effect, that is, it can block NFkB signaling and decrease expression of inflammatory genes [172].

The results of in-vivo studies showed that DnOP caused immunotoxicity in rodents [173,174]. Several studies revealed that DBP and BBP in estrogen receptor (ER) negative breast cancer cells provoked proliferation, invasion, and formation of tumor in breast [175]. The DBP and BBP caused tumor formation in breast by stimulating aryl hydrocarbon receptor (AhR) [176]; thus, activating the downstream cyclic AMP/PKA, CREB1 [176], and HDAC6 signaling pathway [177,178]. The mineralocorticoid receptor (MR) in the interstitial cells of Leydig of adult rat are reduced due to exposure to utero DEHP. This results in the decrease production of testosterone due to alter formation of androgen [67]. In a study it was revealed that in vitro contact of DEHP in male rats, at a dose of 100, 300, and 750 mg/kg/day decreased about 50% of testosterone and aldosterone level while it did not affect the corticosteroids levels [179]. This phenomenon can be described by a decrease in weight of adrenal tissue after a dose of 750 mg/kg/day of DEHP. The weight loss of adrenal tissue is related to decrease levels of angiotensin II receptors. It was noted that there was no significant change in the components renin-angiotensin-aldosterone system (RAAS) in the serum. DEHP is found to be highly toxic in zebra fish with an LC 50 of 0.50 ppm leading to no touch response, tail curvature, embryo mortality, cardiac edema, and necrosis. At a concentration of 1.5 ppm DHP can increase estrogen activity, both in vitro and in vivo [101].

The expression of steroidogenic acute regulatory protein (StAR) mRNA is diminished by DEHP in pregnant mice. This decreases steroidogenesis significantly in both humans and mice. 17α-hydroxylase and cytochrome P450 17A1 are key enzymes in the steroidogenic pathway the in utero mRNA levels of which are lowered by DEHP exposure. The above two situations can take place from either direct exposure of fetal testis or indirect maternal exposure. Aldosterone can activate MR in rat Leydig cells, which potentiates testosterone synthesis by an aldosterone mediated MR mechanism [68].

Phthalates are found to promote allergy by deviating the T-helper 2 (Th2) response and interfere with immunity against infection. Phthalates act on human Plasmacytoid dendritic cells (PDCs) and is involved in the suppression of Interferon-α (IFN-α) and Interferon-β (IFN-β) expression, and hence modulate the capability of T-cell responses [180]. DEHP and BBP have the ability to suppress CpG induced IFN-α/IFN-β expression in PDCs. PDCs are principal cells that secreting type I interferon (IFN), like IFN-α and IFN-β, and are significant in host Th1 responses in immunity against viral infection [181].

DEHP suppressed CpG induced IFN-α/IFN-β appearance in pDCs and the outcome was inverted by aryl hydrocarbon receptor (AHR) antagonist. DEHP suppressed CpG-activated mitogen-activated protein kinase (MAPK)-MEK1/2-ERK-ELK1 and NFҡB signaling pathways. DEHP suppressed CpG-induced interferon regulatory factor (IRF)-7 appearance by suppressing histone H3K4 trimethylation at 1RF7 gene promoter region through inhibiting translocation of H3K4-specific trimethyltransferase WDR5 from cytoplasm into nucleus. BBP or DEHP-treated pDCs suppressed IFN-γ but enhanced IL-13 production by CD4+ T cells [180].

## 5. Phthalate Regulations

Over the few past decades, migration of compounds from food packaging to food has become the main source of putative food toxicity. In fact, PAEs present worldwide concern for human health and environmental risk. In order to harmonize various legislations, and to facilitate and protect consumers, there are several directives and a “threshold policy” implemented by EC and FDA [182].

Framework Directive 89/109/EEC (CEC 1989) establishes two basic principles for food-contact material and articles such as “inertness” and “safety”. The principle of inertness states that any material, article, or its components should be inert enough not to pose any health hazard, unacceptable change in food composition, or deterioration of food qualities. However, Directive 89/109/EEC was repealed by 1935/2004/EC and focuses on general rules for some new topics related to active food-contact materials and safety provisions. Directive 2002/72/EC addresses basic rules and guidelines related to food-contact plastics. This directive is focuses on the materials only made of plastics and plastic gasket in lids (10/2011/EU) and does not consider plastics with other multi-material multilayers [183]. Recently, the latter Regulation 10/11/EU has replaced Directive 2002/72/EC (Commission Regulation No 321/2011) and addresses the use of phthalates in plastics likely coming into contact with food and beverages. The regulation specifically focuses on certain phthalates, listed as toxic for reproduction in annex IV of regulation EU No. 143/2011 EC (CMR category 1B) and states that they are to be completely banned, starting from 1st January 2015. The concerned compounds are BBP, DBP, and DEHP. DnOP, DINP, and DIDP were already prohibited in childcare articles by Directive 2005/84 EC and order 2006-1361 of November 2006.

Consumer’s protection against high exposure to phthalates has been achieved in the EU via the definition of a Candidate List of Substances of Very High Concern (SVHC) because of their endocrine disrupting properties in humans. Moreover, in the USA, they are included in the Priority Toxic Pollutant List of the U.S. Environmental Protection Agency (US EPA). Thirteen phthalates are included in the candidate list of Registration, Evaluation, Authorization and Restriction of Chemicals (REACH). Four of these (DEHP, DBP, BBP, and DIBP) are also on the authorization annex (Annex XIV REACH-ECHA 2009). On the basis of the European Regulation (EC) No. 1907/2006 on the REACH and its amendments (until February 2017), the four phthalates DEHP, BBP, DBP, and DIBP, which are classified as very dangerous substances, shall be produced and sold only after a specific authorization. Furthermore, as resulted from animal studies, European Authorities classified LMW PAEs such as DBP, BBP, and DEHP in Category 1B, namely substances regarded as toxic to reproduction and prohibited for use in toys, children articles, cosmetics, and medical devices [36]. More specifically, toys, and childcare products containing phthalates in a concentration greater than 0.1% of the plasticized material weight shall be marked. DEHP is classified as “priority hazardous substance” under the EU Water Framework Directive and as “toxic to reproduction” in the EU. While there is inadequate evidence in humans for the carcinogenicity of DEHP (IARC Group 3 classification for carcinogenicity), the US EPA has classified DEHP as a Group B2, namely, probable human carcinogen (United States Environmental Protection Agency, 2012). A recent EU risk assessment for DEHP has highlighted the need for more information on the risks for new born babies raised by DEHP contaminated breastmilk. To minimize the health and environmental risk, DEHP has been replaced by DINP and DIDP, which are considered not hazardous according to REACH. HMW PAEs such DINP and DIDP are included in REACH but are not toxic to human health [36].

DINP, DIDP, and DnOP have only been banned in toys and childcare products that children could suck and chew on (1999/815/EC and directive 2005/84/EC). To protect human health, the European Food Safety Authority (EFSA) has set Tolerable Daily Intakes (TDI) for several PAEs: 50 µg kg^−1^ body weight (bw) for DEHP, 10 µg kg^−1^ bw for DBP, 150 µg kg^−1^ bw for DINP and DIDP, and 500 µg kg^−1^ bw for BBP (EFSA 2005 a–e).

According to the regulation No. 10/2011 EC of 14 January 2011 the European Union established limits for many compounds used in packaging and set regulations specifying migration tests using food simulants to determine their probable migration into food. While DIBP is not allowed in food contact materials, EU has set the Specific Migration Limits (SMLs) in plastic food and beverage contact materials for five phthalates, namely DBP, DEHP, BBP, DINP, and DIDP. The SMLs is the Maximum Accepted Concentration (MAC) of a given substance released from a material or article into food and food simulants. For example, the SMLs for DBP, DEHP, and BBP are 0.3 mg Kg^−1^, 1.5 mg Kg^−1^, and 30 mg kg^−1^, respectively, while for DIDP and DINP is 9 mg kg^−1^. Regarding those without SMLs, a limit of 60 mg kg^−1^ in food product is applied. Overall, the plastic packaging must not be released to food simulants more than 10 mg of all compounds in 1 dm^2^ of contact surface between food and packaging (Overall Migration Limit or OML) (Reg 10/11) [7].

Food simulants are used instead of the actual foods in order to simplify the analysis (no matrix effects) and to improve the reproducibility of results [183,184]. Food simulants are usually categorized, based on their chemical properties, as hydrophilic, lipophilic, and amphiphilic. The most commonly used simulants are classified with letters from A to E [185]:

A: Ethanol 10%, simulates hydrophilic foods,
B: Acetic Acid 3%, simulates hydrophilic foods with pH < 4.5,
C: Ethanol 20%, simulates hydrophilic foods with alcohol < 20%,
D1: Ethanol 50%, simulates lipophilic materials, foods with alcoholic contents > 20%, and oil and water in oil emulsions.
D2: Vegetable oil and lipophilic foods.
E: Tenax and dry foods.

DBP and DEHP are only to be used as “Plasticizer in repeated use materials and articles contacting non-fatty foods”; “Technical support agent in polyolefins in concentrations up to 0.05% in the final product”.

DINP, DIDP, and BBP are only to be used as “Plasticizer in repeated use materials and articles”; “Plasticizer in single-use materials and articles contacting non-fatty foods except for infant formulae and follow-on formulae as defined by Directive 2006/141/EC or processed cereal-based foods and baby foods for infants and young children as defined by Directive 2006/125/EC”; “Technical support agent in concentrations up to 0.1% in the final product” [186].

## 6. Occurrence of PAEs in Food

Since food is the major source of exposure to phthalates in humans, it is of great importance to assess toxicological levels of phthalates within it.

First of all, food packaging materials could represent an important source of PAEs in retailed food through migration and leaching. The amount of certain plasticizers that migrate from food contact materials into food is regulated for ESBO (epoxidized soya bean oil) but not for PAEs [187]. Due to their affinity for fat, PAEs are soluble in oil, and therefore they are commonly found in food high in fat. In particular, the latter is expected to be contaminated by HMW PAEs that are more lipophilic. The EC No. 11/2011 states that DEHP, DBP, BBP, DINP, and DIDP are not allowed in the production of fat containing food. According to Cao results, phthalates can migrate into food from plasticized PVC materials such a lid gasket for glass jar, food packaging films-paper, and board packaging (also made from recycled materials) and aluminum foil-paper laminates. Food may be contaminated also during processing and transport, quite often due to the use of PVC gloves in food handling or PVC tubing in olive oil industry or for milking and processing milk. PAEs can migrate during storage from printing inks or adhesives on food wrappers as well as from coatings on cookware that have been contaminated by packaging [188].

In this review we reported the most recent scientific literature of phthalate occurrence in a great variety of food (edible oils and fats, dairy products, meat and poultry, and edible plants) and beverages (alcoholic beverages, soft drink, and water).

### 6.1. Alcoholic Beverages

Alcoholic beverages have high susceptibility to contamination by PAEs as a consequence of their ethanol content (Table 3).

In fact, ethanol may favor the migration of PAEs acting as solvent for PAE extraction. Plasticizers may contact wine during all stages of winemaking: fruit transportation, crush, and storage involve all manner of equipment and materials like pumps, hoses, fining agents, and filtration for final packaging [197].

Del Carlo et al. examined the concentration of various PAEs in 36 commercial red and white wines, 18 wines from local producers, and 8 wines from an experimental pilot plant. Samples were contaminated by DIBP and DEHP at high detection frequency [189]. Commercial wines showed higher detection frequency of total PAEs, DBP, and BBP, than those produced from winemakers and on a pilot plant. No significant influence of the packaging material on the total PAEs content was found. Commercial wines packaged with polyethylene coupled film brick and glass bottles contained significant quantities of DBP, while DBP was found to be under its Limit of Quantification (LOQ) in wines from winemakers and on a pilot plant. The authors suggested that DIBP and DEHP can migrate from the environment during grape growing (plastic foils and laces) as confirmed by the higher content of DIBP in red wines due to the prolonged contact between grape skins and must. On the contrary, DBP and BBP contamination may happen during the winemaking process as a result of migration from materials that could come in contact with wine [189].

In accordance with the previous study, Carrillo et al., found that DBP was the main PAE in ten Spanish wines, followed by DEHP and DEP with total PAE concentrations between 2.7 and 15 µg L^−1^ [189]. However, no statistically significant differences were found in data obtained from different packages (glass bottled with cork and synthetic stoppers, wines in cartons, and wines bag in a box); thus, indicating that contamination occurred prior to packaging. Russo et al. detected DBP, BBP, and DEHP in six commercial wines packed in glass bottles and Tetrapak box and a sample of home-prepared red wine [191]. In addition, 11 commercial wine samples were analyzed and were found highly contaminated [198,199,200].

Noteworthy, Hayasaka analyzed 10 red and white commercial wines from Australia. According to the previous studies, DIBP and DBP were the most widespread, followed by DEHP and BBP in both red and white wines [192].

Chatonnet et al. investigated over 100 commercial French wine samples and 30 grape spirits [193]. DBP, DEHP, and BBP were the most frequently detected compounds. Grape spirit samples were much more contaminated than wine ones, with DBP and DEHP detectable in 90% of samples. Measurable DIBP concentrations were detected in spirits over 20 years old and only rarely in wines. Authors also evaluated PAEs migration from materials that could come in contact with wine and spirit production. The major source of contamination by DBP and DIBP in wines and spirits was the internal coating of wine storage and fermentation vats, made of epoxy resins or polyester-and-glass-fiber. In fact, epoxy resins revealed high level of DBP (0.08%) and DIBP (0.002%). The migration rate was found almost proportional to the storage time. Moreover, the hoses used for pumping contained high concentration of DEHP and DINP and certain synthetic corks presented small quantities of DIBP. The authors also analyzed the plastic bags used to package wines and found small levels of DINP, although in view of the mass and surface of these containers it has not been found the risk of problematic migration. According to the authors, it is advisable for producers to conduct a risk assessment of materials that come in contact with alcoholic beverages; contaminated coatings should be eliminated and the vats should be renovated with modern resins that do not contained undesirable PAEs. Furthermore, several alcoholic beverages such as wine, beer, sangria, and brandy were analyzed [201,202]. The DBP and DEP concentrations were proportional to the ethanol content of the samples. The most highly contaminated sample was the brandy with 65 µg L^−1^ of DBP and 5 µg L^−1^ of DEP.

Jurica et al. carried out a study to determine PAEs contamination in 20 glass bottled plum spirit samples from different Countries of Central and Eastern Europe [193]. The highest concentrations were reported for DBP and DEHP. The authors observed also PAEs migration during five different phases in the plum spirit production process. PAEs were presumably released from the plastic bags used during the plum picking and storing even before the beginning of the production process. At the admission and pureeing phase, DEHP was found to a lesser extent (<20% of the samples). In the final phase of plum spirit production (distillation), mean concentrations of BBP and DEHP increased by 68.8 and 52.9%, respectively, compared to their concentration in the penultimate phase (transfer tank). It seems that the plum distillate, being a more acidic medium, might have drawn out BBP and DEHP from the plastic and rubber components of the pumps or other equipment used during the production phase.

Interestingly, Pellegrino Vidal et al., monitored the occurrence of plasticizers in different kinds of beverages, including red and white wines, beers, and distilled beverage samples with two different analytical methods [194]. One red wine was found to contain high amount of DBP (334 µg L^−1^) above the EU permitted level, while another red wine and a white wine sample were below legislative limits. The irregular red wine showed higher results for DEHP and DEP than the other two samples with 26.8 and 18.2 µg L^−1^ of DEHP. DEP was detected only in the red wine samples (23.6–56 µg L^−1^). Among the alcoholic beverage, beers (Lager and Stout) were found to contain less amounts of the three PAEs. The DBP content was higher for the stout beer, while the DBP concentration was lower for the Lager beer. The DEHP content was similar for the two beers, whereas DEP was detected only in the Stout beer sample. Moreover, the Schnapps and Cachaca samples showed higher amounts of DBP, and DEHP, and DEP were found at high levels in the Cachaca.

A complete historical brandy series (27 years old) was analyzed by Montevecchi et al. The analyzed samples were within the legal limits, except for some very ancient brandies where the higher level of PAEs was probably due to the base wines, to the long ageing and use of plastic pipelines no more operative. An investigation of the repartition of PAEs during the distillation was made in a further study [195]. The concentration of DBP and DEHP in the base wine ranged from 12–79 µg L^−1^ and 5–41 µg L^−1^, respectively. DDBP, having the lowest molecular weight and the lowest boiling point, was entirely carried over into the distillate during the première chauffe and during the distillation of the seconds in the bonne chauffe. The brandy showed DBP and DEHP values of 620 µg L^−1^ and 470 µg L^−1^, respectively. The authors suggested that a rectification step would allow a reduction of PAE concentration in order to reintroduce this valuable fraction cleaner in the distillation process.

Similarly, Plank and Trela suggested that Hazard Analysis and Critical Control Points (HACCP) approach practices such as hazard analysis of critical control points (CCPs) in wine production processes and labelling, to note any specific precautions taken that may help mitigate health risks from plastic additives, effects wine flavor and quality, and ultimately improve consumer confidence, marketability, and wine sales [196].

### 6.2. Mineral Water

Many studies reported the presence of PAEs in bottled mineral water that can be attributed to (i) the quality of the raw material and the technology used in bottle production [203], or perhaps to the chemicals used in the production process [204]; (ii) the use of recycled PET [205]; (iii) the contamination of the water sources with decomposed plastic wastes of dumps [206]; (iv) the cross-contamination in the bottling factory as PAEs are ubiquitous in the environment [207]; (v) the contamination with cap sealing resins [208]; and (vi) the existence of PAEs in the source of water (ground water or tab water). However, according to Bono-Blay et al., PAEs are not relevant contaminants of protected groundwater intended for bottling [209].

Casajuana and Lacorte analysed bottles of mineral water of different trades in PE and PET. DEP, DBP, and DEHP were present at very low initial concentration in both PET and PE bottles, whereas they found increased concentration after storing in PET bottles for 10 weeks up to 30 °C. (Table 4).

According to Keresztes et al., DEHP was the most abundant PAE in PET bottled non-carbonated water followed by DBP and DIBP [210]. The level of PAEs, particularly DEHP, significantly increased during storage at 22, 40, 50, and 60 °C. Moreover, the authors observed the highest PAE concentration in 0.5 L PET containers due to the higher surface/volume ratio.

In another study, the effect of storage time and condition on PAE migration has been investigated [17]. A pronounced increase in the concentration of DEHP, DBP, and BBP was observed at +40 °C after different exposure periods from 24 h to 45 days. On the contrary lower values of increasing concentration for DEHP and DBP was found during freezing condition of 0 °C and −18 °C. In addition to temperature, PAE migration into water resulted to be affected by the duration of exposure. The authors concluded that among the different storage conditions, storage at +40 °C and −18 °C resulted to be the highest and the lowest condition, respectively, responsible of PAE migration. Considering the steady growth of consumption of bottled water and the toxicological effect of PAEs in the field of drinking water, the WHO and EPA set a maximum concentration level (MCL) for DEHP at 6/8 µg L^−1^ [211]. According to these guidelines, exposure to DBP, DEHP, and BBP via consumption of bottled water under condition of common use is well below the MCL stated by WHO and EPA [203] and, in particular, PAE exposure through water intake has been evaluated extremely low for children (0.002–1.1% TDI) [16].

In summary, different conditions such as pH [203,212], storage time [17,207], storage temperature (30–60 °C) [16,213,214], and exposure to sunlight [214] may influence the PAE concentration of PET bottled mineral water. Luo et al., analyzing the frequency of the five targeted phthalates in bottled water of twenty-one countries and more than three hundred different brands, found that the highest concentration of DEHP are detected in bottled water from Thailand, Croatia, the Czech Republic, Saudi Arabia, and China. In bottled waters from Pakistan average levels of BBP, DBP, DMP, and DEP were high. Lou’s study revealed also the phthalates-associated potential risks in both human daily intake and estrogenic effect. According to the authors despite drinking bottle water posed low health concern, the adverse estrogenic effects of phthalates in bottled water from some countries appeared to be significant [215].

Abtahi et al. examining the occurrence of PAEs (DEHP, BBP, DBP, DEP, DMP, and DNOP) in water resources, bottled water, and tap water samples from Tehran Iran, observed that DMP and DEHP were the dominant compounds causing a contribution to the total phthalate levels higher than 60% in all the water sources. In particular, the phthalate levels of drinking water significantly increased by contact of hot water with disposable plastic and paper cups and by sunlight exposure of bottled water. Moreover the authors studied also the health risk of exposure to the phthalates through drinking water and found that drinking water posed a low concern for health determining the hazard quotients (HQs) of DEHP, BBP, DBP, and DEP for all ages both sexes combined Moreover, both the carcinogenic and non-carcinogenic health risks of the phthalates in drinking water were considered to be very low [216].

### 6.3. Soft Drinks

Soft drinks have higher susceptibility to contamination by PAEs than mineral water packed in identical containers [3] (Table 4).

**Table 4:** Occurrence of phthalates in non-alcoholic beverages (µg L^−1^).

Matrix	Phthalate	Average Concentration	Reference
Water in PET bottles	DEHP	0.196	[213]
	DBP	0.046	
	DEP	0.432	
Mineral water	DMP	ND	[217]
	DBP	11.33	
	DEHP	8.79	
Soft drink K sorbate	DMP	759.80	
	DBP	9.00	
	DEHP	36.60	
Soft drink K sorbate and Na benzoate	DMP	500.88	
	DBP	26.75	
	DEHP	15	
Water in PET bottles	DBP	0.21	[212]
	DEP	0.17	
	DEHP	0.02	
Bottled water	DEHP	0.35	[203]
	DBP	0.044	
	DEP	0.033	
Water in PET bottles, non-carbonated	DEHP	0.016–1.7	[210]
	DBP	0.007–0.08	
	DIBP	0.003–0.02	
Water in PET bottles	DEHP	0.217	[17]
	DBP	0.135	
Room temperature	DEHP	0.411	
	DBP	0.116	
Refrigerator	DEHP	0.423	
	DBP	0.124	
Freezing	DEHP	0.317	
	DBP	0.079	
40 °C	DEHP	0.917	
	DBP	0.303	
Mineral water	DEHP	248	[218]
Orange flavored soda	DMP	74	
	DEP	91	
Cola	DMP	105	
	DEHP	1123	
Sport drinks	DEHP	15–98	[219]
Tea	DEHP	16–1263	
Coffee	DEHP	28–159	
Fruit juices	DEHP	22–126	
Espresso coffee surrogates from pre-packed metal capsule	DEHP	220	[1]
	DIBP	240	
	DEP	230	
	DBP	4	
Espresso coffee surrogates from pre-packed plastic capsule	DEHP	1560	
	DIBP	7	
	DBP	7	
Espresso coffee surrogates from pre-packed biodegradable capsule	DEHP	830	
	DIBP	330	
	DBP	120	

Bosnir et al. investigated the migration of DMP, DBP, DEP, BBP, and DEHP in PET-bottled soft drinks and mineral water with different pH and type of preservative used (sodium benzoate and/or potassium sorbate) [217]. They reported that the PAE migration from PET to soft drink was 5 to 40 times higher than mineral water. First of all, phthalate levels found in mineral water free of preservatives were low (20.22 µg L^−1^) as a consequence of the weak acidity (pH = 5.8) of mineral water. The strong acidity (below pH = 3) of soft drinks increased PAE migration; thus, accounting for greater risk. The highest phthalate levels were found in soft drink with K-sorbate (819.40 µg L^−1^), followed by one and a half times lower levels in drinks preserved with Na-benzoate and K-sorbate (116.93 µg L^−1^), seven times lower levels in drinks with Na-benzoate, and nine times lower in drinks preserved with orthophosphoric acid (91.67 µg L^−1^). DMP was found at the highest level of migration into drinks, whereas all other PAEs were measured in levels lower than 19%, and high concentrations of DEHP and DBP has been also observed.

The influence of the type of preservatives and storage times has been also investigated in a study conducted by Ustun et al. They studied the PAE contamination of different brand of beverages taken from different local markets in Turkey. The mean PAE concentration was between 95 and 633 µg L^−1^ in soda (orange flavored), 18 and 1219 µg L^−1^ in lemonade, 19 and 1123 µg L^−1^ in cola, and 85 and 312 µg L^−1^ in mineral water. The level of DMP varied from 74 µg L^−1^ (orange flavored soda) to 105 µg L^−1^ (cola). DEHP showed the highest level of migration into soft drink with average concentration between 248 µg L^−1^ (mineral water) to 1123 µg L^−1^ (cola). DBP was found in concentrations between 91 µg L^−1^ (orange flavored soda) and 367 µg L^−1^ (cola). The total PAE amount also increased with the lengthening of the duration of the duration of soft drinks contamination [218]. In contrast with Bosnir et al. [217], the highest PAE concentrations were measured in soda samples with Na-benzoate and K-sorbate used as preservative. The PAE level in the soda samples preserved with K-sorbate seemed to be similar to samples preserved with Na-benzoate. The authors observed very high concentrations of PAE in mineral water likely due to the presence of preservatives (K-sorbate).

Wu et al. examined different commercial non-alcoholic beverages, including sport drinks, tea drink samples, coffees, and fruit juices purchased in China, and the predominant PAE was DEHP. [219].

Pellegrino Vidal, et al. monitored the PAE content in different alcoholic and non-alcoholic beverages, including mineral and tonic water. DEHP was detected above the allowed MCL (6/8 µg L^−1^) in one sample of mineral and tonic water with concentrations of 12 µg L^−1^ and 14 µg L^−1^. Levels of DEHP were 21 µg L^−1^ in an apple juice sample [202].

In many sport drinks, concentrated juice beverages, tea drinks, jam, jelly, and powder nutraceuticals from Taiwan have been reported to be contaminated by high concentrations of DEHP and DINP. DEHP along with DINP have been illegally used in replacement of the approved clouding agents such as palm oil and Arabic gum, which would normally be added to emulsify the components in the drinks in order to achieve a natural and appealing appearance. The clouding agents made with DEHP could be preserved up to a year differently from those made using palm oil; thus, leading to increased profits. This contamination event has been known as the Taiwan Food Scandal, and consequently many efforts have been made to test the level of phthalates in drinks, and TDI have been developed for different phthalates.

A recent research on the release of PAEs in coffee brewed from pre-packed coffee products was carried out by De Toni et al., which investigated the level of PAEs in coffee prepared using coffee packaged in metal, biodegradable, and two different plastic capsules. DIBP and DEHP were detected in all the surrogates. DIBP and DEHP were the most represented PAEs. Surrogates from biodegradable capsules showed higher concentrations of DBP compared to plastic and metal capsules. DEP was the less represented plasticizer being detected only in surrogates from metal capsule [1].

Recently, phthalate concentrations in 32 commercial tea products (*Camellia sinensis*) from various markets in Naples and on-line shops, were analyzed by Troisi et al. [220]. The most abundant phthalate homologues in the infusions were DBP, DIBP, and DEHP. Despite phthalates are fat-soluble substances and their concentration in water infusions is generally expected to be low, the high temperature of tea infusion preparation can partially overcome the low water solubility. The most likely source of phthalates in commercial tea products seems to be the plastics of the packaging in contact with the tea and/or the tea bag itself. Tea bags are often either made of plastic or have a plastic lining in the case of filter paper-based tea bags.

### 6.4. Edible Oils and Fats

PAEs have been detected in high amount in oily bottled food in a Swiss market survey conducted in 2005 [221]. Levels of DEHP, DINP, DIDP, and DEHA, as a result of migration from PVC gaskets and the material underneath the seal in the closures of glass jars are shown in Table 5.

Marega et al. examined the PAEs contamination level of olives before harvesting, and the presence of phthalates after each production step, in order to define critical points [224]. The authors observed a higher contamination level (DIBP, DBP, and DEHP) in olives collected at mills than in olives directly collected in the olive orchard. These results indicate that the contamination may occur during the harvest and the transport of the olives to the mill. Furthermore, an increase in PAEs levels was observed along the olive oil production chain, probably due to the contact of the olives, paste and oil with pipes, and other plastic materials. However, in most of the cases, contamination levels were lower than the EEC Directive 2007/19/CE suggested limits.

Nanni et al. reported marked differences in the PAEs concentration of vegetable oils sold in Italy deriving from different plant sources (olive, sunflower, peanut, corn, or mixed seeds) and in oils that have undergone different degrees of processing [222]. DINP, known to have replaced DEHP in many industrial applications, was much higher than DEHP, DBP, and DIBP in the edible oils considered in the study. DINP was found at high levels with percentage ranging from 57% (extra virgin olive oil) to 95% (corn oil) of the total PAEs content, followed by DEHP, which was present from 3% in corn oil to 37% in extra virgin olive oil. The olive-derived oils (extra virgin olive oil, olive oil, and olive pomace oil) showed the highest levels of PAEs, being DINP and DEHP, had levels statistically higher in olive pomace oil and in all the three olive-derived oils, respectively. The authors suggested that the phthalate content of oils can decrease during refining (extraction, neutralization, discoloration, and deodorization) so that the oils extracted through pressure are generally more contaminated. The particularly high phthalate content of virgin olive oil was attributed both to the relatively low degree of processing and to the relatively high level of contamination of unprocessed oil derived from a perennial plant (with greater potential for bioaccumulation) in comparison to the ones derived from annual crops. The dietary intakes of DINP, DEHP, and DBP for the Italian consumption of vegetable oils modelled by Nanni et al. accounted for 0.6, 1, and 0.6%, respectively, of TDIs fixed by the EFSA (2005) with any concern for PAE contamination from oil consumption [222].

Bi et al. found 15 plasticizers in 21 edible vegetable oils purchased from a U.S. retail market. DEHP and DIBP were identified in all oil samples [225]. The detection rates for all other plasticizers ranged from 0 to 57.1%. The content of total plasticizers in oil samples was determined to be 210–7558 μg kg^−1^, which was comparable to the content range in oil marketed in Italy. The authors observed a wider range and higher average of total content of plasticizers in olive oil than other oil species (soybean, canola, and corn), indicating the inconsistence of plasticizer contamination from oil packaging and a possible priority for olive oil quality monitoring. DEHP content in two olive oils exceeded relevant SMLs of Europe and China.

In the study conducted by Oh et al., the level of phthalates in different types of oils contained in PET bottles was probably due to the use of adhesives, offset printing inks, and lacquers. DBP was detected in only two olive oil samples (16.7%) at concentrations of 13.2 ± 2.29 and 40.6 ± 2.30 μg kg^−1^. Among the 12 analysed oils, a total of 9 samples (75%) were contaminated with DEHP at slightly high concentrations of 25.0 ± 1.77 (soybean oil) to 806 ± 10.1 μg kg^−1^ (grape seed oil) [226].

According to Lacoste et al., DEHP was observed in several vegetable oils and fats. Some samples such as virgin olive oil, refined grape seed oil, and walnut oil presented DEHP content higher than 1 mg/kg. The authors suggested that chemical refining (low temperature 200 °C vs. 240 °C) conducted to variable elimination of phthalates depending on their molecular weight while physical refining conducted to the total elimination of phthalates (BBP, DEHP, and DIDP) [227].

Sungur et al. investigated the content of phthalates in edible oil sold in Turkish markets. Mean phthalate concentrations were between 102 and 3863 µg L^−1^ in virgin olive oil; 172 and 6486 µg L^−1^ in olive oil; 501 and 3.651 µg L^−1^ in hazelnut oil; 457 and 3415 µg L^−1^ in canola oil; 2227 and 6673 µg L^−1^ in sunflower oil; and 1585 and 6248 µg L^−1^ in corn oil. The highest phthalate levels were measured in sunflower oil, whereas the lowest phthalate levels were determined in virgin olive oil and hazelnut oil. In particular, the highest phthalate levels were determined in oil samples contained in polyethylene terephthalate (PET) [228].

Furthermore, Long-Kai et al. found many highly contaminated edible vegetable oil (i.e., peanut, tea seed, rice bran, sunflower, soybean, corn, rape seed, olive, cotton seed, and wheat germ oils) from China. Total PAE concentration ranged from 40 to 2249 µg kg^−1^. Wheat germ oils were the most contaminated among all tested samples, whereas corn oils were the best varieties. DMP, DBP, and DEHP concentrations in tested wheat germ oils were 90, 21, 290, and 1110 µg kg^−1^, respectively. Five oil samples (one rice bran oil, one peanut oil, two tea seed oils, and one walnut oil) exceeded the MRL 1500 µg kg^−1^ for DEHP set by China. In addition, 13 oil samples (2 rice bran oil, 1 sunflower oil, 2 peanut oils, 2 rape seed oils, 1 cottonseed oil, 2 tea seed oils, 1 wheat germ oil, 1 grape seed oil, and 1 walnut oils) exceeded the MRL 300 µg kg^−1^ for DBP. Among seed oils samples rape seed samples were the most contaminated, while tea seed samples were the best. The authors compared also the effect of packaging material (glass, iron, and PET) on the PAEs content of some samples without finding any high correlation between them [228].

Recently, DEHP and DBP were also detected in four major edible vegetable oil sources from a total of 1016 samples collected throughout China: an edible oil blend, soybean oil, peanut oil, and rapeseed oil. The phthalate with the highest detection rate was DBP (13.48%), followed by DEHP (7.78%). Nevertheless, the two phthalates had the lowest detection rates in soybean oil, which were 1.94% (DEHP) and 5.16% (DBP) [229].

Luo et al. investigated the presence of seven major phthalates in nine different kinds of edible oils (i.e., olive, rapeseed, peanut, sesame, tea seed, corn, soybean, sunflower, and blended oil). DINP, DEHP, DIDP, DBP, DIBP, DEP, and BBP were the main phthalates detected with average concentration of 900, 810, 790, 710, 220, 170, and 100 µg kg^−1^, respectively. The authors revealed the estimated maximum human daily intake (EDI) of DEHP, DBP, BBP, and DIBP through consumption of edible oils were 2.92, 6.79, 1.24, and 1.06 times higher than those via bottled water, respectively. According to the authors edible oils have severe potential adverse estrogenic effects on human 45–396 times of bottled water [230].

### 6.5. Dairy Products

Milk and in particular dairy products have high tendency to be contaminated by phthalates since they are classified as high-fat foods (Table 6).

The contamination can occur along the entire milk production chain from farm to fork [233]. Sharman et al. analyzed the levels of DEHP and total phthalates (expressed as DEHP equivalents) in products (milk, cream, butter, and cheese) from Norway, Spain, and UK [235]. Samples of milk from Norway, obtained at various stage of collection, processing and distribution chain, contained DEHP from 20 to 480 µg kg^−1^ and total phthalates from less than 40 to 5120 µg kg^−1^. The levels of contamination did not increase during the transportation from the storage tank to factory, with DEHP levels of 60–140 µg kg^−1^ being found in both the silo and the tanker. The processing of the milk into products ranging from cream to light milks had the most significant effect on levels of DEHP, whereby the highest levels were found in creams and the lowest levels in the light milk.

Retailed milk and cream samples obtained from Spain resulted to be contaminated with DEHP from less than 10 to 550 µg kg^−1^ and with total phthalate levels from less than 40 to 3040 µg kg^−1^ in cream samples. DEHP appeared to be the predominant contaminant, with over 40% of the total phthalate contribution being attributable to this chemical. This could be explained by the fact that DEHP was the main plasticizer used in food contact materials in Spain. UK pooled milk samples obtained from glass bottles for doorstep delivery contained low levels of DEHP levels (10–90 µg kg^−1^) and the total phthalate (60 to 320 µg kg^−1^) [234].

Retail UK samples of cheese, butter and other fatty products varied considerably in their levels of contamination, the highest being cheese samples containing 17,000 µg kg^−1^ of DEHP and 11,400 µg kg^−1^ total phthalates. However, the majority of samples contained 600–3000 µg kg^−1^ DEHP and 4000–20,000 µg kg^−1^ total phthalates. UK cream samples contained levels of 200–2700 µg kg^−1^ DEHP and 1800–19,000 µg kg^−1^ total phthalates. The levels of phthalate esters observed in UK retail cream and cheese samples were significantly higher than those detected in samples from both Norway and Spain. It is unlikely that the raw milk used for the production of these cheeses was more heavily contaminated, since the levels of DEHP and total phthalates in UK milks was in fact lower than Norwegian one. Sharman et al. suggested that the main route of contamination probably occurs during processing and/or from packaging [231].

Fierens et al. studied the contamination of DMP, DEP, DIBP, DBP, BBP, DEHP, di-cyclohexyl phthalate (DCHP), and DnOP in raw caw milk collected from different Belgian farms [233]. Raw caw milk was found contaminated by DIBP and DEHP due to the ingestion of contaminated feed (i.e., silage and pasture) and, interestingly, the levels of these contaminates changed with seasons. DIBP was detected in winter milk, ranging from 17.2 to 51.5 µg kg^−1^ fat. DEHP levels varied during summer from ND to 787 µg kg^−1^ fat (mean concentration of 400.1 µg kg^−1^ fat) and between 201.3 and 499.7 µg kg^−1^ fat during winter (mean concentration of 298.3 µg kg^−1^ fat). Concentrations of BBP were found in one summer milk sample (15.5 µg kg^−1^ fat) and in four winter milk samples (from 15 to 20.05 µg kg^−1^ fat). The authors revealed that contact materials like PVC tubing during the mechanical milking process had to be considered as additional important contamination points. In fact, DEHP levels increased in the mechanical milking process and cooling tanks, although contamination seemed to be farm dependent. As a result of this study, the decrease of DEHP level in European cows’ milk was also observed because of the substitution of DEHP into the polymers with other types of plasticizers. In contrast, DEHP concentrations in milk outside Europe remain still very high, namely 1410.9 µg kg^−1^ fat on average in milk from South Korea and 5357.2 µg kg^−1^ fat in Canadian milk. Moreover, BBP increased during the mechanical milking process as a result of migration from contact materials.

The contamination of milk and dairy products was investigated at dairy industry and retail level by Fierens et al. [232]. Contamination of these products with phthalates, especially DIBP, DBP, BBP, and DEHP, at some stages of the milk chain was observed. The possible sources of the contamination were labelled as mechanical milking process and intake of the feed by the cattle [232]. The authors revealed that almost no extra phthalate contamination took place during the transportation of milk from the farm cooling tank to the dairy plant cooling tank. During pasteurization, the DEHP content in milk increased from 364 to 426 μg kg^−1^ fat (mean level) and the reason of this increase was most likely due to DEHP containing food contact materials (tubings and sealants). The DEHP migration might have been facilitated by increasing temperature during pasteurization. Once the cooled milk was concentrated, pasteurized, homogenized, and spray dried, the mean DEHP concentration increased from 426 to 476 μg kg^−1^ fat. The milk powder contained also higher level of DIBP and DBP (32 and 28 μg kg^−1^ fat). In addition to those, packaging materials were also identified as another source of contamination. Indeed, DEHP, DIBP, DBP, and BBP were found in packaging materials (can and pouches) used for milk and characterized by a large contact surface. DEHP levels considerably increased in canned milk powder (630 μg kg^−1^ fat); DBP concentrations were 52 and 60 μg kg^−1^ fat in milk powder packed in cans and pouches, respectively, whereas BBP was detected at 53 μg kg^−1^ fat in pouches.

By retail milk examination, DMP and DBP were not detectable on the contrary DIBP increased from ND in raw milk to 18 μg kg^−1^ fat in retail low fat milk, being DEHP mainly detected in retail milk bought in winter. Considering butter packed in foiled paper no specific contamination sources could be detected since the samples were only collected in the beginning and at the end of the milk chain. DEHP was the only detected phthalate with concentration in line with the levels determined in raw summer milk. The same phenomenon was observed for DEHP in cheese. Due to a longer production time, it is conceivable that phthalates present in the products could have been already degraded [233].

According to MeeKyung et al., 15 out of 30 raw bovine milk samples monitored in their study contained DEHP concentrations in raw milk ranging from ND to 154 µg kg^−1^, and the mean concentration was 57 µg kg^−1^. DBP was observed at concentration from ND to 99 µg kg-1 in twenty samples and the mean concentration was 30 µg kg^−1^. The estimated and average intake for a 24-month-old-child is luckily beyond the EU TDI corresponding to the 24% and 8% of TDI respectively [235].

### 6.6. Meat and Poultry

The content of phthalates in thermally processed meat products, after storage at +4 °C in different packages exceeding the limits of the concentration established by EU Commission Regulation 10/2011, was evaluated by Jarosova and Bogdanovicova [236]. DBP and DEHP were ND in the five raw meat samples, whereas there was highly statistically significant evidence of migration of DBP and DEHP depending on the fat content (10 and 50% of fat, respectively) and on the period of storage. The SML for DBP (300 µg kg^−1^) already exceeded after the first day of storage in two samples with 10% of fat, and after the seventh day of storage in one sample. In samples with 50% of fat there was the SML exceeded already after the first day of storage in four samples and after the 14th day of storage in one sample. The concentration of DEHP was comprised from ND to 3570 µg kg^−1^ and from 1260 to 11,670 µg kg^−1^ in meat with 10% and 70% of fat, respectively. In the case of DEHP, in the sample with 10% of fat there was SML exceeded after the first day of storage in one sample, and after the seventh day or 21st of storage in another two samples. The samples with 50% of fat, showed excess of DEHP (1500 µg kg-1 SML) already after the first day of storage. The authors revealed that the DBP content in packages contributed by 20% and, in case of DEHP, by 80% to the overall content of PAEs. They also concluded that the leaching of PAE was 2–21 times higher in samples with 50% of fat than in samples with 10% of fat [236].

Tsai et al. examined the residues of DEHP, BBP, DIDP, DBP, and DINP in unpackaged pork (30 samples) and chicken samples (30 samples) in Taiwan [237]. Thus, eliminating packaging-related contamination, the phthalates detected in the study may have originated from crops cultivated for feed or may have leached from materials in the production process. The major compound detected was DEHP in two pork samples and in three chicken samples. Collectively, 8.33% of the phthalate-residue-containing samples tested positive for DEHP. Although the highest risk of exposure to DEHP was derived from pork consumption, the estimated dietary intake of DEHP residues from both pork and chicken samples was <1% of the TDI value. However, Tsai et al. revealed that the toxicity of phthalates derived from ingesting farmed pork and chickens is not a risk to human health.

### 6.7. Edible Plants

Daily vegetable consumption can pose potential risks to human health since soil PAEs could be taken up and accumulated by plants. As reviewed by Lü et al., PAE compounds in China were widely detected in both urban and agricultural soils as well as in contaminated areas with DBP and DEHP being the predominated compounds. The source identification of PAEs showed that plastic, especially film mulching, is one of the most important sources of PAEs in soil, wastewater irrigation, application of fertilizer, and sewage sludge could also elevate the levels of PAEs in soil [238].

Sun et al., carried out a study to evaluate the uptake and translocation of DEHP, DBP, and their corresponding monoester metabolites by whole plants of lettuce, strawberry, and carrot in order to assess their potential human health risks through dietary intake [239].

The mean PAE concentrations, based on dry plant mass, ranged from 128 to 2391 μg kg^−1^ for DBP and from 654 to 1371 μg kg^−1^ for DEHP in leaves and roots of the three species.

Uptake of both DBP and DEHP was observed in the three plant species, with the overall levels following an order of carrot > strawberry > lettuce. The differences in the uptake of PAEs between plant species may be attributed to the different lipid contents, among other factors. In plant roots, accumulation of DBP (1126−2712 μg kg^−1^) appeared to be greater than that of DEHP in carrot and strawberry, and the concentrations of both DBP and DEHP in roots were significantly higher than those in leaves. Roots, with a higher lipid content than most other plant tissues, may preferentially accumulate hydrophobic compounds. In addition to the higher log Kow (log Kow = 4.45 for DBP and 7.50 for DEHP), DEHP has lower water solubility than DBP and so the plant uptake is lower than DBP. The mean bioconcentration factor (BCF) values of the leaf or root of the three species ranged from 0.26 to 4.78 for DBP and 1.31 to 2.74 for DEHP. The BCF values of DBP in roots of strawberry and carrot were larger than those of DEHP, whereas the BCF values of DBP in leaves were smaller than those of DEHP.

Sun et al., also observed uptake of MBP and MEHP in the three plant species. The MBP concentrations in both leaves and roots of carrots were slightly higher than the others. The concentration of MEHP was also higher in carrot leaves, while the root of lettuce showed the highest MEHP accumulation. In addition, concentrations of MBP in leaves and roots of all three plants species were consistently higher than those of MEHP, and the difference may be attributed to their physicochemical properties, such as Kow and pKa [239]. Once they have been taken up, PAEs are readily transformed into their monoesters. Incubation of PAEs and monoalkyl phthalate esters (MPEs) in carrot cell culture showed that DBP was hydrolyzed more rapidly than DEHP, while the monoesters were transformed more quickly than their parent precursors.

In conclusion according to Sun et al., food plants may accumulate PAEs as a result of the large use of plastic films in agricultural production. Plastic films are extensively used as surface mulch, soil tarps after fumigation and row covers. In addition, the use of plastic greenhouses serves many functions, such as extending the growing season, conserving water, controlling weeds, and maintaining high quality produce. The estimated amounts of plastic mulch films and greenhouse covers are 0.7 and 1.0 million tons per year, respectively.

Results from this study clearly demonstrated that human exposure calculated using the whole plant data was well below the reference doses for individual PAEs. However, given the extensive metabolism of PAEs to monoesters in both whole plants and plant cells, metabolites such as MPEs should be considered when assessing human exposure via consumption of vegetables grown in PAE-contaminated soils [219,239,240].

Chen et al. investigated PAE contamination levels in vegetables both sold on the market and grown in greenhouses. Vegetables growing in greenhouse agriculture had higher DBP and DEHP content than those growing in open fields [240]. Moreover, there was more accumulation of PAEs in vegetables leaf compared to PAEs in soils. The concentrations of DEHP, DIBP, and DBP in the air inside the greenhouses were much higher than those outside. These results suggested that vegetables may absorb PAEs not only through their roots from soil but also through their leaves from air. The mean concentrations of DIBP, DBP, and DEHP were no significantly higher in vegetables growing in greenhouses covered with plastics than in open fields.

Chen et al. revealed that the mean concentrations of total PAEs in the vegetables from the markets were slightly higher than those in leaves of vegetables from greenhouses, such as bokchoy, eggplant, green bean, green pepper, and tomato, in which more DIBP and DBP were detected. The total concentration of PAEs in vegetable leaves from greenhouses and vegetables sold on the market were in the ranges of 1580–8090 and 950–6360 µg kg^−1^ (fresh weight), respectively.

The results of statistical analysis showed that the concentration of DEHP positively correlated with greenhouses cultivation time, suggesting that DEHP may be derived from plastic films, while DBP from fertilizer and pesticides. People in northern cities in China had higher exposure of PAEs from vegetables than those in southern cities. In conclusion, the high detection frequency of PAEs in vegetables sold on the Chinese market indicates that exposure pathway of PAEs to humans through vegetable consumption should be of concern in cumulative risk assessments. A special attention should be given to individuals who work in greenhouses due to high DEHP concentration inside greenhouse air [240].

## 7. Conclusions

PAEs are ubiquitous compounds and food contaminants that became of great concern a few decades ago, when they started to be regarded as a global threat for human health. In particular, LMW PAEs have shown highly endocrine-disrupting properties (European Union Risk Assessment Report 2003) and have been classified as harmful substances in Europe and in REACH. Since then, many efforts have been made by the European Commission (EU) and the United States Environmental Protection Agency (ES EPA) to regulate and limit their distribution and application in different industrial fields, including food contact materials. Although there exist a threshold policy establishing Specific Migration Limits (SMLs) and Tolerable Daily Intake (TDIs) for individual phthalates per person, it is not so easy to estimate their contribution and discriminate them among other environmental pollutants to which people are simultaneously exposed every day.

According to the Agency for Toxic Substances and Disease Registry (2002) the average daily human exposure to DEHP is about 0.003–0.03 mg/kg/day (7.7–77 μmol/kg/day) with children being the most vulnerable subjects due to the ability of PAEs to penetrate into placenta, excreted into breast milk, and used in the fabrication of toys [84].

Because of the noncovalent binding, PAEs can easily leach out of the various matrices; thus, entering the food and other commodities. Once ingested, DEP is absorbed as a monoester and transformed into lipophilic xenobiotic chemicals that are likely responsible for health dysfunctions. These compounds become unsuitable part of dietary intake as a possible consequence of accidental contamination during food processing and packaging, agrochemical treatments (wastewater irrigation, sewage sludge disposal, and film mulching), leached pesticides, chemical industrial waste, or inappropriate transport of goods. In the attempt to monitor their concentration along the food chain, many studies have been carried out to detect the extent of phthalates in a great variety of food (edible oils and fats, dairy products, meat and poultry, and edible plants) and beverages (alcoholic beverages, soft drink, and water). What emerged from all the studies is that the migration of these chemicals from packaging into food depends on the type of packaging materials (polyethylene terephthalate; polyvinylchloride; gaskets of lids for glass jars; and carton), the high-fat composition of food, the ethanol content, the pH of the medium, the degree of lipophilicity, and biodegradation processes. Noteworthy, it has been evaluated that the concentration of these compounds seems to increase along the food chain, from the animal or vegetal sources to the distributed food products, i.e., dairy products (milk, cheese, and butter) [232,233], oils [230], and wines [196]. Interestingly edible plants can pose cumulative risk for both animal and human consumption [240].

In the present review, we examined PAEs contamination in foodstuffs and beverage from an analytical and toxicological point of view, highlighting the molecular mechanisms underlying their putative involvement in several human diseases, as well as the critical aspects of the contamination raised by the different type of food matrices, packaging, storage time, pH influence, and temperature variations [210,236].

It has to be emphasized that in defiance of the need to limit PAEs distribution, it is really complicated to substitute them as plasticizers for their excellent characteristics (i.e., increasing flexibility, durability, and workability). Hence, it is of fundamental importance to identify advanced processing technologies able to ameliorate the quality of food packaging, so to minimize chemical migration and contamination of food, drinks, oils, and other consumer products.

The greatest challenge remains the difficulty in harmonizing various legislations in different Countries as well as standardizing test conditions and methods of human biomonitoring.

This review increases the body of knowledge regarding on PAEs impact on human health and highlights the importance of studying the role of these chemicals in the onset and progression of many human pathological conditions. Many of their toxic effects (i.e., reproductive and developmental) are mediated through the interaction with xenosensing receptors, although they would also activate receptor-independent signaling pathways that have been correlated to various diseases, such as cancer (i.e., breast, skin, and liver), endometriosis, infertility, sex anomalies, asthma, hypertension, type II diabetes, obesity, nephron- and hepato-toxicity, as well as neurological disorders [241]. It is plausible that phthalates are not the only players in the onset or progression of these pathologies, but they may give a huge contribution as additional risk factors together with other environmental contaminants.

So far, it is still an open question whether PAEs are positively [42,56,242], negatively [243,244], or in any way [245] associated with the levels of reproductive hormones and fertility in general, being epidemiological studies quite controversial and inconsistent about it. As it is now recognized that environmental chemical exposure during fetal development may induce diseases in the adult life, more studies at various life stages are needed in order to establish a biologically plausible causal relationship between PAEs exposure and the induced adverse effects; thus, helping to assess the risks on clinical and public health.

It should be underlined that PAEs-mediated effects on reproductive system may largely vary depending on PAEs exposure (low vs. high), wherein low-doses level were consistent with changes in steroidogenesis pathway (stimulation of testosterone production and prevention of infertility) [62,246] whereas higher-level inverted this effect [247]. This antipodal behavior of PAEs associated to their non-monotonic effects in spermatogenesis and male fertility makes it difficult to estimate the risk following human exposure. Another critical aspect is linked to the choice of the study design, often recruiting individuals with confounding abnormalities regarding reproductive system or genetic polymorphisms [248]. If in vitro or in vivo studies may not represent realistic conditions regarding the doses of single or mixed PAEs used in the experiments, epidemiological studies appear often limited and not generalizable to the general population because of the non-representative sample size, which would restrict the statistical power of the findings and would not allow an effective prediction. Therefore, additional larger population studies will be of great help to further identify not only the individual weight of PAEs in human health but also the sum with other compounds, having in mind that in real-life scenarios, it is impossible to control people exposure to a wide variety of chemicals from the numerous sources [249], and that these chemicals nowadays coexist in natural environments. Noteworthy, many endocrine disruptors, such as PAEs and Bisphenol-A, can act together in a synergistic way to produce additive effects in the human body [250].

In view of the growing evidence conferring to epigenetic mechanisms a key role in PAEs-mediated effects in reprotoxicity, tumorigenesis, and metabolic diseases, the evaluation of the risk correlated to PAEs exposure through high throughput in silico computational analysis, enabling the integration of data from urine or blood with genomics, and the establishment of a dose-response relationship, would elucidate the effective role of PAEs in the onset of endocrine, metabolic and neurological disorders, among others.

In conclusion, more studies should be addressed in order to overcome crucial issues related to heterogeneous methodologies, short-term studies, lack of human samples, and few matrices, and to better understand the impact of phthalates on human health; thus, protecting consumers from hazardous chemicals in foodstuffs and put in place more health-protective regulations.

## Figures and Tables

**Table 1:** Chemical structure of common phthalates and their main applications.

Chemical Name	Structure	Use
Diethyl phthalate (DEP)	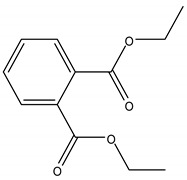	Personal care products (e.g., fragrances), coatings (e.g., pharmaceuticals), dyes, pesticides
Di-(2-ethylhexyl) phthalate (DEHP)	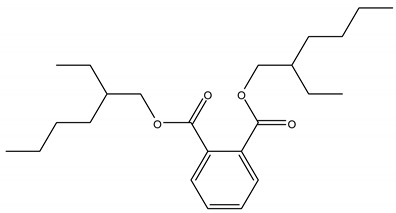	Perfumes, PVC plastics used in household products (e.g., toys, floor tiles and furniture upholstery, cables, garden hoses, wall coverings, and gloves), food packaging, blood storage bags, and medical devices
Di-isononyl phthalate (DINP)	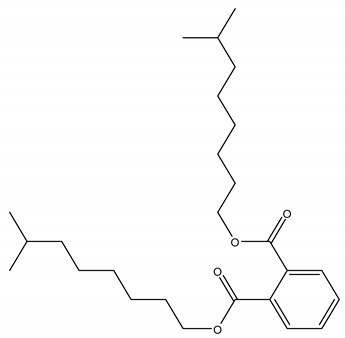	Plasticizer, remaining in rubbers, inks, adhesives and sealants, paints, and lacquers
Benzyl Butylphthalate (BBP)	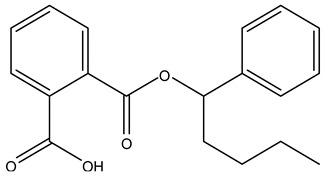	Vinyl flooring, adhesives and sealants, car-care products, toys, food packaging, synthetic leather, industrial solvents, glues, personal care products, and automotive products
Di-n-butyl phthalate (DBP)	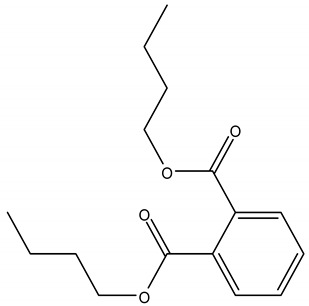	Cellulose acetate plastics, solvent for oil-soluble dyes, pesticides, personal care products (e.g., nail polish and cosmetics), lacquers, varnishes, and coatings (e.g., pharmaceuticals)

**Table 2:** List of abbreviations of the most common parent phthalates and their metabolites.

Phthalate	Abbreviation	Formula
Benzyl-butyl Phthalate	BBP	C19H20O4
Di-butyl Phthalate	DBP	C16H22O4
Di-cyclohexyl Phthalate	DCHP	C20H26O4
Di-ethyl Phthalate	DEP	C12H14O4
Di-etylhexyl Phthalate	DEHP	C24H38O4
Di-isobutyl Phthalate	DIBP	C16H22O4
Di-isodecyl Phthalate	DIDP	C28H46O4
Di-isononyl Phthalate	DINP	C26H42O4
Di-methyl Phthalate	DMP	C10H10O4
Di-n-octyl Phthalate	DnOP	C24H38O4
Mono-n-butyl phthalate	MnBP	C12H14O4
Monobenzyl phthalate	MBzP	C15H12O4
Monocarboxy-isononly phthalate	MCNP	C18H24O6
Monocarboxyoctyl phthalate	MCOP	C₁₇H₂₂O₆
Mono-(3-carboxypropyl) phthalate	MCPP	C12H12O6
Mono(2-ethyl-5-carboxypentyl) phthalate	MECPP	C16H20O6
Mono(2-ethyl-5-hydroxyhexyl) Phthalate	MEHHP	C16H22O5
Mono(2-ethylhexyl) Phthalate	MEHP	C16H22O4
Mono(2-ethyl-5-oxohexyl) Phthalate	MEOHP	C16H20O5
Mono-ethyl phthalate	MEP	C10H10O4
Monoisobutyl Phthalate	MiBP	C12H14O4
Monoisononyl Phthalate	MINP	C1_7_H2_4O4_
Mono-methyl Phthalate	MMP	C9H8O4
Mono-methyl Phthalate	MNOP	C9H8O4

**Table 3:** Occurrence of phthalates in alcoholic beverages (µg L^−1^).

Matrix	Phthalate	Average Concentration	Reference
Wines in glass bottle	DIBP	0.099	[189]
	DBP	0.053	
	DEPH	0.076	
	BBP	0.040	
Wines in polyethylene coupled film brick	DIBP	0.076	
	DBP	0.115	
	DEHP	0.078	
Wine in glass bottles with one-piece cork	DEP	4.22	[190]
	DBP	2.21	
	BBP	4.29	
Wine in glass bottles with synthetic stoppers	DEP	2.95	
	DBP	1.02	
	DEHP	5.22	
Wines in cartons	DBP	2.22	
	DEHP	3.90	
Wines in bag-in-box	DMP	0.61	
	DEP	1.78	
	DBP	0.30	
Commercial white wines in tetrapak box	DBP	10.0	[191]
	BBP	1.0	
	DEHP	16.0	
Commercial red wines in glass bottles	DBP	7.3–23	
	BBP	0.1–5.2	
	DEHP	3.1–15.8	
Commercial white wines in glass bottles	DBP	19.3–21.3	
	BBP	0.4–7.0	
	DEHP	9.2–15.1	
Home-prepared red wines	DBP	22.8	
	BBP	ND	
	DEHP	2.4	
Commercial red wine samples (Australia)	DIBP	5.3–10.7	[192]
	DBP	3.4–9.3	
	DEHP	1.7–4	
	BBP	3.5–6.3	
Commercial white wine samples (Australia)	DIBP	4.6–9.1	
	DBP	2.9–3.7	
	DEHP	2.3–4	
	BBP	0.3–1.1	
Commercial wines (France)	DBP	0.273	[193]
	BBP	0.008	
	DEHP	0.134	
Commercial spirits (France)	DBP	0.314	
	BBP	0.026	
	DEHP	0.513	
	DIBP	0.103	
Glass bottled plum spirits	DBP	414.5	[194]
	DEHP	423.8	
	BBP	79.0	
	DIBP	38.8	
Red wine samples	DBP	334.0	[195]
	DEHP	80.3	
	DEP	56	
Stout beer	DBP	74.7	
	DEHP	16.6	
	DEP	4.7	
Lager beer	DBP	1.1	
	DEHP	18.2	
	DEP	ND	
Schnapps	DBP	76.6	
	DEHP	28.0	
	DEP	4.7	
Cachaca	DBP	40.5	
	DEHP	140.0	
	DEP	25.8	
Base wine samples	DBP	12.0–79.0	[196]
	DEHP	5.0–41.0	
Brandy samples	DBP	620.0	
	DEHP	470.0	

**Table 5:** Occurrence of phthalates in vegetable oils from different plant sources (µg Kg^−1^).

Oil sources	Phthalate	Average Concentration	Reference
Extra virgin olive	DEHP	1134	[222]
	DINP	1722	
	DBP	90	
Olive	DEHP	1262	
	DINP	2884	
	DBP	360	
Sunflower	DEHP	134	
	DINP	971	
	DBP	35	
Various seed	DEHP	132	
	DINP	1361	
	DBP	30	
Corn	DEHP	81	
	DINP	2982	
	DBP	23	
Peanut	DEHP	334	
	DINP	1518	
	DBP	41	
Soybean	DEHP	77	
	DINP	1017	
	DBP	22	
Olive pomace	DEHP	1643	
	DINP	6480	
	DBP	224	
Peanut oil	DEHP	1250	[223]
	DBP	250	
Teaseed oil	DEHP	1250	
	DBP	1610	
Rice bran oil	DEHP	650	
	DBP	1060	
Sunflower oil	DEHP	260	
	DBP	140	
Soybean oil	DEHP	140	
	DBP	60	
Corn oil	DEHP	100	
	DBP	20	
Rapeseed oil	DEHP	160	
	DBP	470	
Cottonseed oil	DEHP	350	
	DBP	270	
Olive oil	DEHP	850	
	DBP	110	
Wheat germ oil	DEHP	1110	
	DBP	21,290	
Grape seed oil	DEHP	930	
	DBP	1690	
Walnut oil	DEHP	1590	
	DBP	1206	
Sesame	DEHP	290	[223]
	DBP	80	
Corn	DEHP	150	
	DBP	170	
Rapeseed	DEHP	400	
	DBP	90	
Teaseed	DEHP	140	
	DBP	20	
Soybean	DEHP	190	
	DBP	120	

**Table 6:** Occurrence of phthalates in dairy products (µg kg^−1^).

Matrix	Phthalate	Average Concentration	Reference
Milk	DEHP	20–480	[231] Milk samples from Scandinavian countries
Milk in silo and tanker	DEHP	60–140	
Cream 35% fat	DEHP	1060–1670	
Milk <1% fat	DEHP	20–40	
Milk	DEHP	10–40	Milk samples from Spain
Cream	DEHP	480–550	
Milk	DEHP	10–90	Milk from UK
Cheese	DEHP	600–3000	
Cream	DEHP	200–2700	
Summer milk	DEHP	ND–787.6	[232]
	DIBP	ND–15	
	DBP	ND–15.3	
	BBP	ND–15.5	
Winter milk	DEHP	201.3–499.7	
	DIBP	17.2–51.5	
	DBP	ND–15	
	BBP	10–20.5	
Milked by hand	DEHP	<60	
	DIBP	29	
	DBP	<15	
	BBP	<10	
Milked by machine	DEHP	123.5	
	DIBP	15.1	
	DBP	ND	
	BBP	14.3	
Raw milk cooling tank	DEHP	364	[233]
	DIBP	<15	
	DBP	ND	
	BBP	ND	
Pasteurized milk cooling tank	DEHP	426	
	DIBP	ND	
	DBP	ND	
	BBP	<15	
Milk powder before filling	DEHP	478	
	DIBP	32	
	DBP	28	
	BBP	ND	
Milk after filling (can)	DEHP	630	
	DIBP	56	
	DBP	52	
	BBP	12	
Milk after filling (pouch)	DEHP	523	
	DIBP	31	
	DBP	60	
	BBP	53	
Milk powder at retail (can)	DEHP	566	
	DBP	75	
	DBP	53	
	BBP	12	
Milk powder at retail (pouch)	DEHP	526	
	DIBP	75	
	DBP	53	
	BBP	12	
	DBP	80	
	DBP	170	
Milk samples	DEHP	57	[234]
	DBP	30	
